# Deep learning versus manual morphology-based embryo selection in IVF: a randomized, double-blind noninferiority trial

**DOI:** 10.1038/s41591-024-03166-5

**Published:** 2024-08-09

**Authors:** Peter J. Illingworth, Christos Venetis, David K. Gardner, Scott M. Nelson, Jørgen Berntsen, Mark G. Larman, Franca Agresta, Saran Ahitan, Aisling Ahlström, Fleur Cattrall, Simon Cooke, Kristy Demmers, Anette Gabrielsen, Johnny Hindkjær, Rebecca L. Kelley, Charlotte Knight, Lisa Lee, Robert Lahoud, Manveen Mangat, Hannah Park, Anthony Price, Geoffrey Trew, Bettina Troest, Anna Vincent, Susanne Wennerström, Lyndsey Zujovic, Thorir Hardarson

**Affiliations:** 1 Virtus Health, Sydney, New South Wales Australia; 2 IVFAustralia, Sydney, New South Wales Australia; 3 Unit for Human Reproduction, 1st Dept of Ob/Gyn, Medical School, Faculty of Health Sciences, Aristotle University of Thessaloniki, Thessaloniki, Greece; 4 Centre for Big Data Research in Health, Faculty of Medicine and Health, University of New South Wales, Sydney, New South Wales Australia; 5 Melbourne IVF, Melbourne, Victoria Australia; 6 School of BioSciences, University of Melbourne, Parkville, Victoria Australia; 7 School of Medicine, Dentistry and Nursing, University of Glasgow, Glasgow, UK; 8 TFP Fertility, Institute of Reproductive Sciences, Oxford, UK; 9 Vitrolife, Viby J, Denmark; 10 Vitrolife, Gothenburg, Sweden; 11 Virtus Health, Melbourne, Victoria Australia; 12 TFP Fertility, Nottingham, UK; 13 IVIRMA Global Research Alliance, Livio Gothenburg, Gothenburg, Sweden; 14 Queensland Fertility Group, Brisbane, Queensland Australia; 15 The Fertility Unit, Horsens Hospital, Horsens, Denmark; 16 Aagaard, Aarhus, Denmark; 17 Dept of Reproductive Medicine, Sahlgrenska University Hospital, Gothenburg, Sweden; 18 TFP Fertility, Southampton, UK; 19 Imperial College London, London, UK; 20 The Fertility Unit, Aalborg University Hospital, Aalborg, Denmark

**Keywords:** Medical research, Translational research

## Abstract

To assess the value of deep learning in selecting the optimal embryo for in vitro fertilization, a multicenter, randomized, double-blind, noninferiority parallel-group trial was conducted across 14 in vitro fertilization clinics in Australia and Europe. Women under 42 years of age with at least two early-stage blastocysts on day 5 were randomized to either the control arm, using standard morphological assessment, or the study arm, employing a deep learning algorithm, intelligent Data Analysis Score (iDAScore), for embryo selection. The primary endpoint was a clinical pregnancy rate with a noninferiority margin of 5%. The trial included 1,066 patients (533 in the iDAScore group and 533 in the morphology group). The iDAScore group exhibited a clinical pregnancy rate of 46.5% (248 of 533 patients), compared to 48.2% (257 of 533 patients) in the morphology arm (risk difference −1.7%; 95% confidence interval −7.7, 4.3; *P* = 0.62). This study was not able to demonstrate noninferiority of deep learning for clinical pregnancy rate when compared to standard morphology and a predefined prioritization scheme. Australian New Zealand Clinical Trials Registry (ANZCTR) registration: 379161.

## Abstract (web summary)

A randomized controlled trial evaluating the selection of a single blastocyst for transfer by deep learning did not demonstrate noninferiority in clinical pregnancy rates when compared to trained embryologists using standard morphology criteria.

## Main

Artificial intelligence (AI), specifically deep learning, has been heralded as a revolutionary technology, with its potential in healthcare, particularly in relation to the interpretation and reporting of medical images, widely acknowledged^1^. Despite its potential, the clinical utility of AI in healthcare remains underexplored in randomized controlled trials (RCTs)^2^.

During in vitro fertilization (IVF), a key challenge is selecting the embryo that will give the patient the best chance of a live birth from a group of usable embryos. Currently, during the culture period, the embryologist removes the embryos from the incubator, usually more than once, and performs a ‘snapshot’ morphological assessment under a microscope^3^. This approach is time consuming, subjective and has, in essence, changed little since the first IVF birth 45 years ago^4,5^.

The advent of time-lapse incubators, capable of capturing frequent embryo images over five to six days of growth, has circumvented the need to remove embryos from the incubator for assessment, minimizing disturbance^6^. Combining this with a validated automated selection algorithm has the additional potential to substantially improve workflow efficiency compared to standard incubation and to overcome subjectivity^7^. To date, several studies have employed deep learning algorithms for embryo quality grading or development stage classification based on static images from time-lapse incubators^8–11^. Recently, the deep learning algorithm iDAScore was developed that uses spatial (morphological) and temporal (morphokinetic) patterns from time-lapse images across the first six days of embryological development (to the blastocyst stage) to predict, with high discriminative ability and reproducibility, the probability of a transferred embryo implanting and progressing to a clinical pregnancy^12,13^.

Despite numerous proposed applications of AI in IVF, their integration into routine clinical practice hinges on robust evidence demonstrating their efficacy compared to traditional methods^14^. A number of studies of AI have suggested significant improvements in consistency of embryo scoring^15,16^, but these evaluations have been retrospective. Two previous studies^17,18^ have prospectively examined the efficacy of traditional machine learning algorithms based on previous data and human input for embryo selection. By contrast, deep learning is a more advanced form of machine learning that enables computers to learn from experience and understand the world in terms of a hierarchy of concepts^19^. Because the computer gathers knowledge from experience, there is, unlike conventional machine learning, no need for a human operator to formally specify any of the knowledge needed by the computer. No RCTs of deep learning in embryo selection have previously been reported. Despite the potential utility of deep learning, there is a need to demonstrate noninferiority to standard embryologist assessment of the embryos.

We therefore undertook a prospective randomized noninferiority trial to determine whether selection of a single blastocyst for transfer by deep learning results in a clinical pregnancy rate comparable with a 5% inferiority margin to that achieved by trained embryologists using standard morphology criteria.

## Results

**Fig. 1:**
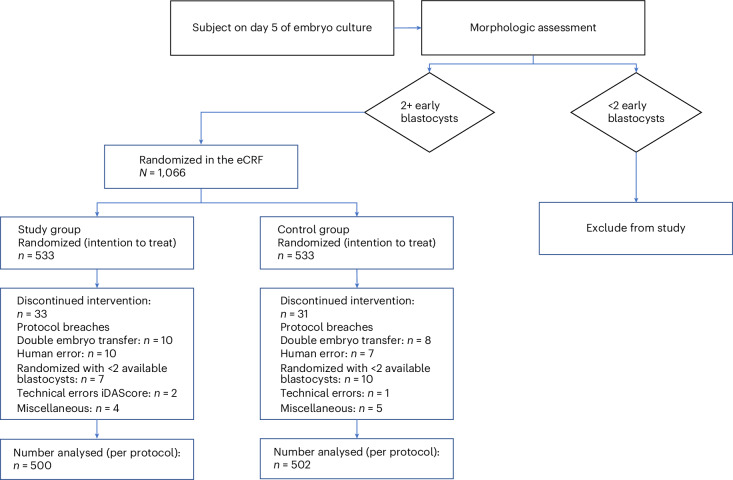
Patient flow diagram. The number of patients randomized in the electronic case report form (eCRF) to the study group and the control group then, following exclusions for discontinued intervention, the number analyzed per protocol.

From 16 January 2020 until 30 September 2022, a total of 1,751 participants were eligible, with 1,066 patients included in the trial (533 in the study arm and 533 in the control arm). Of these, 1,002 participants were included in the per-protocol (PP) analysis after the exclusion of 64 participants, owing to protocol violations (Patient flow diagram; Fig. 1). The demographics and clinical characteristics of the patients at the time of randomization were well balanced between the trial groups, except from the type of transfer, with an extra 7.5% of patients undergoing a frozen–thawed embryo transfer in the study group compared to the control group (Table 1).

**Table 1:** Baseline and treatment characteristics (intention-to-treat population)

Variable	Study group (*n* = 533)	Control (*n* = 533)
Maternal age	33.7 (3.7) 34 (22; 43)	33.8 (3.8) 34 (20; 42)
Reason for infertility (couple)		
No clinical subfertility	44 (8.3%)	42 (7.9%)
Unexplained infertility	206 (38.6%)	202 (37.9%)
Known cause for infertility	283 (53.1%)	289 (54.2%)
Maternal body mass index	25.4 (4.9) 24.3 (15.1; 44.6)	25.5 (5.0) 24.4 (15.6; 48.9)
Number of previous stimulated IVF cycles leading to oocyte pick-up	0.533 (1.008) 0 (0; 9)	0.606 (1.103) 0 (0; 11)
Subjects with a previous pregnancy in current relationship	146 (27.4%)	155 (29.1%)
FSH treatment total dosage	2,426 (1206) 2,163 (56; 7,500)	2,393 (1231) 2,200 (77; 6,750)
GnRH downregulation		
Agonist	65 (12.2%)	65 (12.2%)
Antagonist	468 (87.8%)	468 (87.8%)
Number of oocytes	14.8 (8.0) 13 (2; 52)	14.7 (8.6) 13 (2; 76)
Method of fertilization		
Standard IVF	226 (42.4%)	227 (42.6%)
ICSI	278 (52.2%)	276 (51.8%)
Combined	29 (5.4%)	30 (5.6%)
Number of normally fertilized oocytes (2PN)	9.02 (5.20) 8 (1; 34)	9.01 (5.35) 8 (1; 44)
Number of blastocysts at Gardner grade 2 or beyond by day 5	5.26 (3.70) 4 (0; 28)	5.03 (3.53) 4 (0; 29)
Number of cryopreserved embryos on day 5 and 6	4.80 (3.86) 4 (0; 28)	4.08 (3.72) 3 (0; 31)
Gardner grade on day 5 of the transferred embryo^a^		
1 or 2	21 (4.0%)	15 (2.8%)
3AA, 4AA, 5AA, 6AA	320 (61.1%)	361 (68.5%)
Others	183 (34.9%)	151 (28.7%)
iDAScore of the transferred embryo	9.09 (0.68) 9.3 (4.7; 9.9)	
Type of transfer		
Fresh (current randomized cycle)	320 (60.3%)	353 (66.4%)
After cryopreservation or total freeze	211 (39.7%)	179 (33.6%)

For categorical variables *n* (%) is presented.

For continuous variables mean (s.d.)/median (minimum; maximum) is presented.

^a^Freeze-all embryos were selected on information up to day 6, which, in some cases, led to an embryo graded 1 on day 5 being prioritized.

FSH, follicle-stimulating hormone; GnRH, gonadotropin-releasing hormone; ICSI, intracytoplasmic sperm injection.

### Primary outcome

**Fig. 2:**
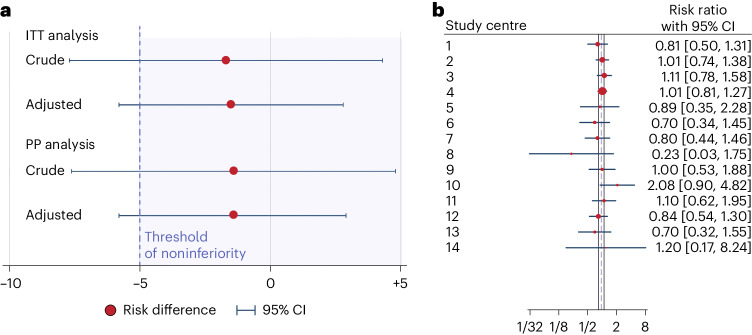
Non-inferiority analysis and risk ratio per center. **a**, Crude and adjusted risk difference (red circle represents mean risk difference and blue error bars represent 95% CI of the risk difference) in clinical pregnancy rates between the iDAScore and the control groups (a negative risk difference indicates lower clinical pregnancy rate in the iDAScore group) per intention-to-treat (ITT) analysis (*n* = 1,066) and PP analysis (*n* = 1,002). **b**, Risk ratio (red circle represents mean risk ratio and blue error bars represent 95% CI of the risk ratio) of clinical pregnancy rate for each participating center (a ratio >1 indicates better results in the iDAScore group).

The primary outcome, clinical pregnancy, occurred in 248 of 533 patients (46.5%) in the study group and in 257 of 533 patients (48.2%) in the control group. Noninferiority of embryo selection using the deep learning algorithm was not shown, with an absolute risk difference of −1.7 percentage points (95% confidence interval (CI), −7.7, 4.3) and a rate ratio of 0.96 (95% CI, 0.85, 1.10) (Table 2 and Fig. 2a). The results were similar for the PP analysis, full analysis set (FAS) and the primary efficacy analysis with adjustment for center and selected allocation variables (Table 2 and Supplementary Tables 1 and 2).

**Table 2:** Primary and adjusted efficacy analysis on clinical pregnancy with fetal heartbeat after the first embryo transfer

Population	Study group^a^	Control^a^	*P* value^b^	Crude difference^c^ (95% CI)	Crude risk ratio (95% CI)	Adjusted^d^ difference (95% CI)	Adjusted^d^ risk ratio (95% CI)
Intention to treat	248 of 533 (46.5%) (42.2%–50.9%)	257 of 533 (48.2%) (43.9%–52.6%)	0.62	−1.7 (−7.7; 4.3)	0.96 (0.85; 1.10)	−1.5 (−5.8; 2.8)	0.97 (0.88; 1.06)
Per protocol	237 of 500 (47.4%) (43.0%–51.9%)	245 of 502 (48.8%) (44.4%–53.3%)	0.66	−1.4 (−7.6; 4.8)	0.97 (0.85; 1.10)	−1.4 (−5.8; 2.9)	0.97 (0.88; 1.07)

^a^For categorical variables proportion, percentage and exact 95% CI is presented.

^b^For comparison between groups, Fisher’s exact test (2-sided) was used.

^c^Confidence limits calculated using Farrington–Manning test.

^d^Adjusted for each woman’s age, number of previous stimulated IVF cycles leading to oocyte pick-up, number of oocytes, fertilization method, number of 2PN oocytes and center.

### Secondary outcomes

Results similar to the primary outcome were observed for the secondary outcomes of the positive human chorionic gonadotropin (hCG) rate, nonviable intrauterine pregnancies and ongoing pregnancy rate (Supplementary Tables 3 and 4). Live birth rate was similar between the two groups where the live birth rate for the iDAScore group was 39.8% (212 of 533 patients) and 43.5% (232 of 533 patients) in the standard morphology criteria group (risk difference −3.9%; 95% CI, −9.9, 2.2; *P* = 0.24) (Supplementary Tables 3 and 4).

**Fig. 3:**
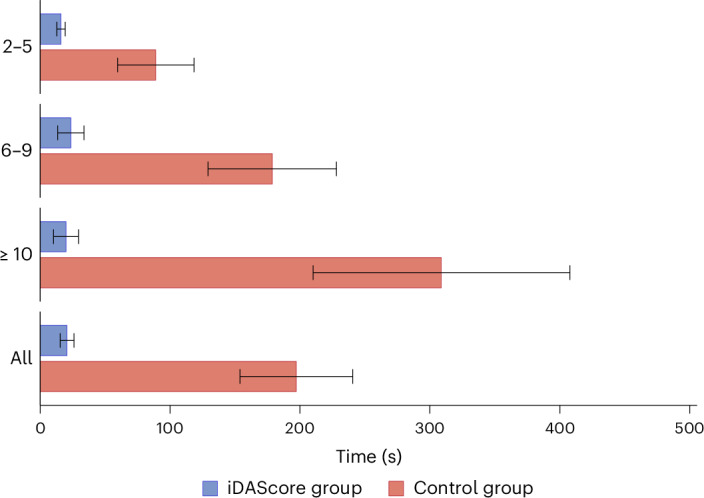
Time use for day 5 evaluation in the iDAScore group and control group. Mean and standard deviation for all patients (*n* = 38) and for embryo cohort sizes from 2–5 (*n* = 9), 6–9 (*n* = 16) or 10 or more (*n* = 13) in the treatment.

The study group was found to have an almost 10-fold reduction in the time required for evaluation, with a mean standard deviation time of 21.3 ± 18.1 seconds in comparison to the control group, which took 208.3 ± 144.7 seconds (*P* < 0.001) regardless of the number of embryos available on day 5 (Fig. 3).

### Safety

No medical adverse events were recorded, while adverse device effects were reviewed and recorded as protocol breaches.

### Sensitivity analyses

A prespecified sensitivity analysis, by adjusting for each woman’s age, the number of previous stimulated IVF cycles leading to oocyte pick-up, the number of oocytes obtained, the fertilization method used, the number of normally fertilized oocytes and the center, did not materially change the results obtained (Table 2).

Assessment of the efficacy of the deep learning algorithm compared to standard morphology criteria in each of the 14 study centers showed broadly similar outcomes, with risk ratios (RR) for clinical pregnancy ranging from 0.23 to 2.08, with all centers having wide CIs that included unity (Fig. 2b).

In the prespecified subgroup analysis where the results in only women >35 years of age were compared, clinical pregnancy occurred in 85 of 228 (37.3%) in the study group and in 89 of 228 (39.0%) in the control group, with a risk difference between the groups of −1.8 (95% CI, −11.1, 7.6). The positive hCG rate, the nonviable intrauterine pregnancy, ongoing pregnancy rate and live birth rate were also similar between the study and control groups (Supplementary Table 5). In a further prespecified interaction analysis, we observed differing performance between the study and control groups in fresh-embryo transfer and freeze-all cycles (RR, 1.08 versus RR, 0.81; *P* = 0.022).

To assess the moderating potential of variables including maternal age and the number of available blastocysts, prespecified interaction analyses were performed in the FAS. Neither maternal age nor the number of available blastocysts had any significant interaction with the clinical pregnancy effect sizes obtained in the groups compared (Supplementary Table 6). Clinical pregnancy rates were comparable in fresh cycles, with the study and control groups resulting in 48.1% (154 of 320) and 44.5% (157 of 353) (*P* = 0.35). By contrast, the freeze-all cycles showed a significant difference (*P* = 0.032) with the study and control groups resulting in 49.5% (94 of 190) and 61.3% (100 of 163) (Supplementary Table 7). The baseline demographics (Supplementary Table 8) within these subgroups revealed no significant imbalances.

Pregnancy follow-up (Supplementary Table 9) showed no statistically significant difference in pregnancy outcomes for the two groups. There was no significant difference in the male-to-female ratio in the study group compared to the control group (53.5% versus 58.6%, *P* = 0.32; Supplementary Table 9).

We investigated concordance of embryo selection and found that, of the 523 participants in the study group, the deep learning algorithm and standard morphology selected the same embryo for transfer in 344 participants (65.8%), while in 179 participants, different embryos were selected by the two techniques. The clinical pregnancy rates in these two groups were 48.3% and 44.7% (Supplementary Table 10). The Gardner grades for the embryos in each group are shown in Supplementary Table 11.

### Post hoc analyses

Additional analysis was performed to investigate any difference in laboratory outcomes of participants who underwent a fresh transfer and those who underwent a frozen transfer after a freeze-all cycle (Supplementary Tables 12 and 13). The participants who underwent a frozen transfer had a significantly higher number of oocytes obtained, 20.4 (19.3–21.4) in the freeze-all group, compared to 11.7 (11.3–12.0) in the fresh-transfer group. This resulted in the freeze-all group having significantly more day 5 blastocysts: 7.0 (6.6–7.5) in the freeze-all group and 4.2 (4.0–4.4) in the fresh-transfer group. The Gardner grades for the embryos in each group are shown in Supplementary Table 13.

## Discussion

This RCT rigorously evaluated deep learning in embryology laboratories. The principal finding was that this study was not able to demonstrate noninferiority of deep learning in terms of clinical pregnancy rates when compared to standard morphology and a predefined prioritization scheme. However, the study did demonstrate that deep learning, as exemplified by the iDAScore, significantly accelerates evaluation times compared to standard morphology-based embryo selection.

Before this study, the performance of AI algorithms for blastocyst transfer and their impact on clinical pregnancy outcomes had not been directly compared to standard morphological criteria used by embryologists in a prospective RCT setting. Most existing studies have primarily focused on retrospective analyses of AI’s capability to objectively grade embryos and blastocysts. A recent systematic review^7^ only identified three studies that report the association with live birth rate^20–22^. Each of these studies was considerably smaller than the current trial (175 to 458 patients), used locally derived datasets with internal validation and were not RCTs^20–22^.

Previously, a machine learning algorithm, used adjunctively with morphology, trained to predict blastocyst development potential on day 3 of embryo development was tested prospectively in a previous multicenter study by Kieslinger et al.^17^. No difference in ongoing pregnancy rate was observed when using this algorithm compared to using standard morphology. The Kieslinger study highlights one of the challenges in performing clinical studies. The study was registered in 2015, but blastocyst stage transfer is now routinely performed by most clinics. Similarly, the known implantation data score (KIDScore), a morphokinetic algorithm requiring manual evaluation of embryos, has been prospectively evaluated^18^. No difference in ongoing pregnancy rates between KIDScore and standard morphology were reported, with no notable workflow efficiency due to the manual input requirement.

Our study, using a deep learning algorithm in combination with time-lapse, diverges from these approaches by assessing blastocyst development without the need for manual inputs, thus reducing evaluation time. In combination with the use of time-lapse incubation systems, deep learning embryo assessment offers the potential for minimizing time and risks associated with handling and moving embryos in the laboratory^23^. However, potential laboratory efficiency gains from deep learning are only a component of the costs of IVF and have to be considered within the context of formal cost-effectiveness studies of the complex health economics of this emerging technology.

Although the pregnancy rates were clinically similar between the two groups, we could not conclude noninferiority because the lower bound of the CI surpassed our predetermined noninferiority margin of −5%. The study design of noninferiority was selected as the primary scientific objective of our study to evaluate whether the automated selection of a single blastocyst for transfer by the deep learning algorithm (iDAScore) yields a clinical pregnancy rate comparable to that achieved by trained embryologists using standard morphology criteria and a predefined prioritization scheme.

An important deviation from the predefined hypothesis was the unexpectedly higher pregnancy rates (48.2%) in the control group, which significantly exceeded the anticipated rate of 35.4%, calculated from retrospective data from a population meeting the entry criteria to this study, used for the sample size calculation. This deviation negatively impacted on the power of this trial to conclude noninferiority. The higher pregnancy rates observed in both groups, surpassing typical rates reported in US, European and Australian national datasets^24^, may be a result of the participation in an RCT environment (the Hawthorne effect^25^). For example, a similar prospective trial assessing the efficacy of freezing all embryos^26^ observed similar elevated pregnancy rates. The higher pregnancy rates observed could also be an outcome of the rigorous morphological assessment protocol employed. As part of our trial design, we standardized embryo selection across participating centers, using a study-specific prioritization scheme (detailed in the Supplementary Information), based on the Gardner grading scheme^27^. This standardization, whether through AI or a uniform morphological assessment protocol, suggests potential for enhancing outcomes compared to current variable practices. This finding underscores the importance of consistency in embryo evaluation methodologies^4^, which has consistently been shown by AI on static images and time-lapse sequences^8–13^, and hints at the potential benefits of integrating standardized approaches in IVF procedures.

Regardless of the cause of the higher pregnancy rates observed, future trials to assess an effect of this magnitude, assuming similar control group pregnancy rates and trial parameters (5% noninferiority margin, true difference of −1.7%, 90% power, *α* = 0.05 and *β* = 0.10) would require an impractically larger sample size to demonstrate noninferiority, estimated at around 7,800 participants^28^. The inability of a practically sized trial to detect a small but clinically important effect of this sort sets a challenge for the future design of RCTs.

We observed an inconsistency in the performance of the deep learning model between fresh- and frozen-embryo transfers. In contrast to the fresh-embryo transfers, where the iDAScore group had a 3.7% higher clinical pregnancy rate, embryo selection by the deep learning model significantly underperformed compared to the control in the frozen-embryo group. This finding was surprising as previous studies based on retrospective data have found a significantly better iDAScore ranking in thawed-blastocyst data in older women^29^ and thawed-euploid transfers^30^. The reason for the disparity is unclear. In the freeze-all cases, there were more embryos to choose from, and this may be a factor in the difference or it may be speculated that elements of the basis of iDAScore analysis preferentially selected embryos with a predisposition to a poorer freeze–thaw performance. Finally, it is possible that the outcome observed in this trial for frozen embryos could be attributable to chance alone as this was an observational post hoc analysis. It should be noted that the clinical pregnancy rate in the fresh transfers in the control group was 44.5%, whereas the frozen-embryo transfers in the same group had a remarkably higher clinical pregnancy rate of 61.3%. Further investigation into the factors influencing outcomes in frozen-embryo transfer is warranted.

While live birth is normally perceived as the definitive outcome in studies of assisted reproduction, this study used clinical pregnancy as the primary outcome, while reporting live birth as a secondary outcome. This was on the basis that the deep learning system was specifically trained on clinical pregnancy^12,13,29,31^ and the aim of the trial was to test whether iDAScore achieves noninferiority in the endpoint on which it had been trained. However, analysis of the live birth data did not materially alter the conclusion reached by the trial.

Recently, several authors have expressed concerns about possible biases introduced by AI concerning sex ratios^32^. For example, Ueno et al.^31^ observed a nonsignificant increase in the male ratio with increasing iDAScore on a large retrospective live birth dataset. However, this was not confirmed in our prospective study, where no significant difference was found in the male-to-female ratio.

Another ethical concern when using deep learning for embryo selection is the black-box nature of such models^32^. Some studies have investigated explainability by introducing so-called heat maps to show where and when a deep learning network focuses when generating a score^16^. However, the clinical value of such approaches needs further studies. Currently, most studies on explainability have investigated the correlation between well-established morphological and morphokinetic parameters and the output from deep learning models^13,30^. These studies have found a strong correlation between iDAScore and manual embryo morphology and morphokinetics, suggesting that the deep learning models directly or indirectly focus on image features in a way similar to that done by embryologists. This study did not add to the understanding of how AI interprets embryogenesis. However, ongoing improvements in AI methodologies, coupled with interdisciplinary research efforts, will gradually enhance our collective knowledge of embryogenesis, ultimately contributing to the refinement of assisted reproductive technologies.

It is important to acknowledge several limitations in our trial. First, iDAScore was derived and tested solely within the context of the EmbryoScope incubator, limiting its generalizability to other time-lapse incubator systems. Second, the time-to-pregnancy was not assessed, as only the first embryo was prioritized for transfer, leaving an equivalent number of embryos available for future use in both groups. Similarly, we have not reported cumulative live birth rates because that would require transfer of all embryos, although we anticipate this to be similar as no embryos were deselected for use based on the iDAScore. As we had underestimated the time required for standard morphological criteria assessment, a smaller substudy than planned was required to show the observed time differences. Last, the continued evolution of deep learning algorithms^33^ presents a challenge for ongoing evaluation via traditional RCTs, suggesting the necessity for alternative research methodologies in assessing future iterations^34^.

The present randomized trial examined the efficacy of using a deep learning algorithm for the selection of which embryo to transfer for couples undertaking assisted conception. This study was unable to demonstrate noninferiority in clinical pregnancy rate to standard morphology. However, the deep learning approach studied did provide a consistent user-independent approach with a 10-fold reduction in assessment time.

## Methods

### Trial design and oversight

This randomized, noninferiority, double-blind parallel-group controlled trial was carried out at 14 IVF clinics in Australia and Europe. Written informed consent was obtained from all participants. Each participating clinic’s respective Human Research Ethics Committee reviewed and approved the trial protocol, which had been previously registered with the Australian New Zealand Clinical Trials Registry (ANZCTR) registration: 379161). All the data in the study were collected using a commercial eCRF provider MariaDB v.10.6 and Database Platform VI v.7.1.6 by Stockholm Data Design, Sweden. The randomization was performed within the eCRF using web-based interactive response technology. The statistical analysis plan and detailed protocol are available in the Supplementary Information. A steering committee, responsible for trial design and the manuscript’s first draft, supervised the trial. They affirm the data completeness and accuracy and the trial’s fidelity to the protocol. An independent data safety and monitoring board provided consistent oversight for the trial’s conduct. The trial was implemented using EmbryoScope incubators and received funding from the manufacturer, Vitrolife, Denmark.

### Study population

The study was conducted between January 2020 and September 2022. We included women undergoing IVF or ICSI after ovarian stimulation with gonadotrophins and gonadotrophin releasing hormone analogs and with the intention to treat by either transfer of a single fresh embryo on day 5 or, in a freeze-all cycle, the first rewarmed embryo. Inclusion criteria required women to be before their 42nd completed birthday with at least two early blastocysts on day 5 of embryo culture, the day of randomization. We excluded participants if they were involved in treatment using donated eggs, intended to perform preimplantation genetic testing, used additional laboratory interventions such as intracytoplasmic morphologically selected sperm injection, polarized light microscopy, assisted hatching or had previously participated in the trial.

Ovarian stimulation and triggering of final oocyte maturation was performed under each site’s standard protocol, supervised by the treating physician. After oocyte retrieval, all embryos were fertilized by either IVF or ICSI based on individual clinical decisions and incubated at 5% oxygen in the EmbryoScope+ time-lapse incubator system (Vitrolife) until day 5 or 6.

Provided at least two embryos reached the early blastocyst stage (grade 2 according to Gardner grading)^18^ or beyond at 114–118 hours post insemination (hpi) (day 5 of embryo culture), patients were eligible for randomization. The embryos were assessed using standard morphology criteria (Gardner grade)^18^ previously demonstrated in a similar population^18^. To ensure that the embryologist was blinded to the randomization at the time of morphology selection, the highest-grade blastocyst was selected for transfer, using the prioritization strategy detailed in the clinical investigation protocol in the Supplementary Information, before randomization. Once this selection had been performed and documented, the participant was then randomized through a web-based interactive response technology (MariaDB v.10.6 and Database Platform VI v.7.1.6 by Stockholm Data Design) to ensure allocation concealment.

If the patient was randomized into the control arm, the blastocyst selected on morphological grounds was transferred and iDAScore was not performed. If the patient was randomized into the study arm, iDAScore v.1.2 was performed to generate a score for each embryo and the blastocyst with the highest iDAScore was then selected for transfer.

The iDAScore v.1 algorithm was trained and evaluated based on a large dataset from 18 IVF centers consisting of 115,832 embryos, of which 14,644 were transferred embryos with known outcome^13^. The algorithm is based on an inflated three-dimensional convolutional neural network where the output is fed to a bidirectional long short-term memory. The input to the model was 128 images of the central focal plane and a resolution of 256 × 256 pixels. The sequence was offset by 12 hpi, thus effectively covering 12 to 140 hpi. The v.1 iDAScore was used throughout the study period and no clinic-specific or other adjustments were applied during the course of the study.

The trial-group assignment was performed in an unblinded manner to the embryologist, but both the treating clinician and the patient remained blinded to the randomization outcome until after the first embryo transfer. Any remaining embryos were cryopreserved if they met the normal freezing criteria for the laboratory or, in addition, in the study arm if they had reached Gardner grade 3 or beyond and had an iDAScore of 5 or more. All remaining embryos were re-evaluated on day 6, rescored at 138–142 hpi and assessed using the Gardner grade and iDAScore. In both groups, the prioritization of frozen embryos for subsequent warming and transfer was based on all information up to day 6. All blastocysts were transferred in EmbryoGlue (Vitrolife). Embryo transfer and luteal support were performed and administered according to each clinic’s standard protocol.

### Time taken to evaluate the embryos for transfer

We compared the time taken to evaluate the same embryos across three laboratories using the two assessment techniques. We initiated a timer from the moment of opening the patient file in the EmbryoScope+ software until the selection of the embryo for transfer, using either iDAScore or standard morphology criteria.

### Outcomes

The primary outcome for this study was the achievement of clinical pregnancy after the first embryo transfer, whether fresh or frozen–thawed in freeze-all cycles. We defined clinical pregnancy as an intrauterine gestation with a fetal heartbeat observed after 7–9 weeks gestation. Secondary outcomes included positive hCG rate, nonviable intrauterine pregnancies, ongoing pregnancy rate (at 12 weeks gestation) and live birth rate. Nonviable intrauterine pregnancy was defined as a pregnancy in which an intrauterine gestation was seen with no fetal heartbeat found. Live birth was defined as delivery of at least one liveborn child.

### Statistical analyses

The noninferiority margin for this study was established at −5% for clinical pregnancy rate. On the basis of existing evidence from participating clinics, we expected the clinical pregnancy rate following the first embryo transfer to be 35.4%. To demonstrate with 90% power (*α* = 0.05 and *β* = 0.10) that the lower limit of the two-sided 95% CI for the difference between the iDAScore and the standard morphology criteria group would not be less than −5%, with an expected increase in clinical pregnancy of 5% or more in the iDAScore group, we required 494 women per group. This figure was increased to 520 women per group to account for a potential 5% of loss to follow-up.

For the time taken to evaluate embryos for transfer substudy, the standard deviation for the difference between the morphological (Gardner grade) and iDAScore assessment was estimated as 60.6 seconds from a pilot study of 20 patients. To find a mean difference in times between Gardner grade and iDAScore of 45 seconds with a two-sided Fisher’s nonparametric permutation test for paired observations, on significance level 0.05, with a power of 80% we required a minimum of 51 patients. The substudy was performed in three clinics and, to ensure a representation of clinical practice, different embryo cohort sizes, ranging from small (2–5 embryos), medium (6–10 embryos) to large (>10 embryos), were included.

The primary efficacy analysis was performed in the intention-to-treat population (denoted as the ITT in the clinical investigation protocol and statistical analysis plan in the Supplementary information), including all patients who underwent randomization and had an embryo transfer. All randomized subjects with measurement of the primary efficacy value were included in the FAS. We conducted complementary analyses on the PP population. Planned and prespecified sensitivity analyses, adjusting for significant a priori specified covariates using multivariable logistic regression were performed. These covariates included each woman’s age (<25, 25 to <30, 30 to <35, 35 to <40 and ≥40), number of previous stimulated IVF cycles leading to oocyte pick-up (0 or >1), number of oocytes (2 to 5, 6 to 10, 11 to 15, 16 to 20, 21 to 25 and >26), fertilization method (IVF or ICSI), number of two pronuclear oocytes (2 to 5, 6 to 10, 11 to 15, 16 to 20, 21 to 25 and >26) and center. We also undertook a prespecified subgroup analysis for the older female age groups (>35 years) and an interaction analysis according to each woman’s age and the total number of embryos available for selection. Last, we compared the performance of the iDAScore algorithm at each individual center.

For the time taken to evaluate embryo substudy, we used Fisher’s nonparametric permutation test for paired observations and estimated the differences between the morphological and iDAScore with varying embryo numbers. All the statistical analyses were executed using SAS v.9.4, with the support of N.-G. Pehrsson of Statistika Konsultgruppen. Additional details are provided in the Methods section of the Supplementary Appendix.

### Role of the funding source

The project was funded by a grant from Vitrolife. As sponsor, employees of Vitrolife Group participated in the study design and writing of the report. Data collection and analysis were performed by independent entities.

### Reporting summary

Further information on research design is available in the Nature Portfolio Reporting Summary linked to this article.

## Online content

Any methods, additional references, Nature Portfolio reporting summaries, source data, extended data, supplementary information, acknowledgements, peer review information; details of author contributions and competing interests; and statements of data and code availability are available at 10.1038/s41591-024-03166-5.

## Supplementary information

Supplementary Information Supplementary Tables 1–13, the clinical investigation protocol and the statistical analysis plan.

Reporting Summary

## Data Availability

Data collected for the study, including deidentified participant data and the data dictionary, will be made available to others. Related documents, including the study protocol, the statistical analysis plan and the informed consent forms will be available following publication. The data and documents will be made available on request to academic researchers following review by the study’s steering committee (P.J.I., C.V., D.K.G., S.M.N., J.B., M.G.L. and T.H.) and completion of a data sharing agreement. Requests for data sharing should be addressed to the corresponding author. Approval of requests for academic purposes will be provided within 3 weeks and the data supplied following approval. Approval will not be provided for commercial use of the data.

## Code availability

The iDAScore algorithm is commercially available. It was developed by Vitrolife to be used within the EmbryoViewer workstation with EmbryoScope time-lapse incubators. The code is proprietary, but iDAScore and the necessary hardware can be obtained from Vitrolife (www.vitrolife.com).
