# Supplementary material for: Deep learning versus manual morphology-based embryo selection in IVF: a randomized, double-blind noninferiority trial
Source: Nat Med. 2024 Aug 9;30(11):3114–20. doi: 10.1038/s41591-024-03166-5 (PMC11564097; doi:10.1038/s41591-024-03166-5)
Supplement: Supplementary file 1 — Supplementary Tables 1–13, the clinical investigation protocol and the statistical analysis plan. [file 41591_2024_3166_MOESM1_ESM.pdf]

nature medicine

Supplementary information https://doi.org/10.1038/s41591-024-03166-5

Deep learning versus manual morphology-based embryo selection in IVF: a randomized, double-blind noninferiority trial

In the format provided by the authors and unedited

Table S1 Complementary Analysis of Primary Efficacy Analysis (FAS Population)

Variable	Study group (n=510)	Control (n=516)	p-value	Difference between groups Mean (95% CI)	Rate Ratio (95% CI) for Clinical pregnancy
Clinical pregnancy with fetal heartbeat after the first embryo transfer	248 (48.6%) (44.2%-53.1%)	257 (49.8%) (45.4%-54.2%)	0.71	-1.2 (-7.3; 4.9)	0.98 (0.86; 1.11)

For categorical variables n (%) and exact 95% CI is presented.
For comparison between groups Fisher´s Exact test (2-sided) was used for dichotomous variables.
Confidence Limits calculated using Farrington-Manning.

Table S2. Primary Efficacy Analysis Sensitivity Analyses, adjusted for center and selected allocation variables (FAS Population)

Variable	Study group (n=510)	Control (n=516)	Difference between groups Mean (95% CI)	Adjusted Rate Ratio (95% CI) for Clinical pregnancy
Clinical pregnancy with fetal heartbeat after the first embryo transfer	248 (48.6%) (44.2%-53.1%)	257 (49.8%) (45.4%-54.2%)	-0.9 (-5.5; 3.6)	0.98 (0.89; 1.08)

For categorical variables n (%) and exact 95% CI is presented.
For comparison between groups linear mixed effects regression model (with identity link function) was used with center as a random effect and fixed effects for selected allocation variables.
Adjusted Rate Ratio and CI calculated using Poisson regression model with a robust error variance.
Adjusted for Woman's age, Number of previous stimulated IVF cycles leading to oocyte pick-up, Number of oocytes, Fertilization method, Number of 2PN oocytes and center.

Table S3 Secondary Efficacy Analysis (ITT Population)

Variable	Study group (n=533)	Control (n=533)	p-value	Difference between groups Mean (95% CI)	Rate Ratio (95% CI) for Clinical pregnancy
Positive hCG rate, per randomized patient	297 (55.7%) (51.4%-60.0%)	303 (56.8%) (52.5%-61.1%)	0.76	-1.1 (-7.2; 4.9)	0.98 (0.88; 1.09)
Non-viable intrauterine pregnancies, per randomized patient	49 (9.2%) (6.9%-12.0%)	46 (8.6%) (6.4%-11.3%)	0.83	1.7 (-8.8; 12.3)	1.07 (0.73; 1.56)
Ongoing pregnancy rate[1], per randomized patient	229 (43.0%) (38.7%-47.3%)	241 (45.2%) (40.9%-49.6%)	0.50	-2.3 (-8.3; 3.8)	0.95 (0.83; 1.09)
Livebirth rate, per randomized patient	212 (39.8%) (35.6%-44.1%)	232 (43.5%) (39.3%-47.9%)	0.24	-3.9 (-9.9; 2.2)	0.91 (0.79; 1.05)

[1]Patients with a viable pregnancy at ≥12 weeks of gestation
For categorical variables n (%) and exact 95% CI is presented.
For comparison between groups Fisher´s Exact test (2-sided) was used for dichotomous variables.
Confidence Limits calculated using Farrington-Manning.

Table S4 Secondary Efficacy Analysis (PP Population)

Variable	Study group (n=500)	Control (n=502)	p-value	Difference between groups Mean (95% CI)	Rate Ratio (95% CI) for Clinical pregnancy
Positive hCG rate, per randomized patient	285 (57.0%) (52.5%-61.4%)	288 (57.4%) (52.9%-61.7%)	0.95	-0.4 (-6.6; 5.9)	0.99 (0.89; 1.11)
Non-viable intrauterine pregnancies, per randomized patient	48 (9.6%) (7.2%-12.5%)	43 (8.6%) (6.3%-11.4%)	0.58	3.1 (-7.6; 13.9)	1.12 (0.76; 1.66)
Ongoing pregnancy rate[1], per randomized patient	219 (43.8%) (39.4%-48.3%)	232 (46.2%) (41.8%-50.7%)	0.45	-2.4 (-8.7; 3.8)	0.95 (0.83; 1.09)
Livebirth rate, per randomized patient	204 (40.8%) (36.5%-45.3%)	223 (44.4%) (40.0%-48.9%)	0.25	-3.7 (-10.0; 2.6)	0.92 (0.80; 1.06)

[1]Patients with a viable pregnancy at ≥12 weeks of gestation
For categorical variables n (%) and exact 95% CI is presented.
For comparison between groups Fisher´s Exact test (2-sided) was used for dichotomous variables.
Confidence Limits calculated using Farrington-Manning.

Table S5 Clinical pregnancy, Positive hCG rate and Non-viable intrauterine pregnancies - Women older than 35 years (ITT Population)

Variable	Study group (n=228)	Control (n=228)	p-value	Difference between groups Mean (95% CI)
Positive hCG rate, per randomized patient	111 (48.7%) (42.0%-55.4%)	107 (46.9%) (40.3%-53.6%)	0.78	1.8 (-7.9; 11.4)
Clinical pregnancy with fetal heartbeat after the first embryo transfer	85 (37.3%) (31.0%-43.9%)	89 (39.0%) (32.7%-45.7%)	0.77	-1.8 (-11.1; 7.6)
Non-viable intrauterine pregnancies, per randomized patient	26 (11.4%) (7.6%-16.3%)	18 (7.9%) (4.8%-12.2%)	0.27	3.5 (-2.3; 9.4)
Ongoing pregnancy rate[1], per randomized patient	77 (33.8%) (27.7%-40.3%)	81 (35.5%) (29.3%-42.1%)	0.77	-1.8 (-10.9; 7.4)
Livebirth rate, per randomized patient	69 (30.3%) (24.4%-36.7%)	76 (33.3%) (27.3%-39.9%)	0.55	-3.1 (-12.1; 5.9)

[1]Patients with a viable pregnancy at ≥12 weeks of gestation

For categorical variables n (%) and exact 95% CI is presented.
For comparison between groups Fisher´s Exact test (2-sided) was used for dichotomous variables.

Table S6 Exploratory Interaction Analyses (FAS Population)

	Baseline variables<br>Interaction with Treatment group<br>p-value		
Variable	Maternal age	Number of blastocysts at Gardner Grade 2 or beyond by day 5	Freeze all
Positive hCG rate, per randomized patient	p=0.7185	p=0.8865	p=0.0403
Clinical pregnancy with fetal heartbeat after the first embryo transfer	p=0.9200	p=0.3915	p=0.0215
Non-viable intrauterine pregnancies, per randomized patient	p=0.7708	p=0.0459	p=0.5002
Ongoing pregnancy[1], per randomized patient	p=0.7416	p=0.1700	p=0.0052
Livebirth, per randomized patient	p=0.9617	p=0.4822	p=0.0315

[1]Patients with a viable pregnancy at ≥12 weeks of gestation.

Analyses performed with no other adjustments with Poisson regression model with a robust error variance and without multiplicity correction.

Table S7 Exploratory Interaction Subgroups Analysis (FAS Population)

Variable	Study group	Control	p-value	Difference between groups Mean (95% CI)	Rate Ratio (95% CI) for Clinical pregnancy
Fresh (current randomized cycle)	n = 320	n = 353			
Positive hCG rate	187/320 (58.4%)	193/353 (54.7%)	0.35	3.8 (-3.8; 11.4)	1.07 (0.94; 1.22)
Clinical pregnancy rate	154/320 (48.1%)	157/353 (44.5%)	0.35	3.7 (-3.9; 11.2)	1.08 (0.92; 1.27)
Ongoing pregnancy rate[1]	143/320 (44.7%)	143/353 (40.5%)	0.28	4.3 (-3.4; 11.9)	1.10 (0.93; 1.31)
Livebirth rate	129/320 (40.3%)	138/353 (39.1%)	0.75	1.3 (-6.4; 9.0)	1.03 (0.86; 1.24)
After cryopreservation/total freeze	n = 190	n = 163			
Positive hCG rate	110/190 (57.9%)	110/163 (67.5%)	0.078	-10.2 (-20.9; 0.6)	0.86 (0.73; 1.01)
Clinical pregnancy rate	94/190 (49.5%)	100/163 (61.3%)	0.032	-11.9 (-22.4; -1.5)	0.81 (0.67; 0.97)
Ongoing pregnancy rate[1]	86/190 (45.3%)	98/163 (60.1%)	0.0056	-14.8 (-25.2; -4.4)	0.75 (0.62; 0.92)
Livebirth rate	83/190 (43.7%)	94/163 (57.7%)	0.010	-13.9 (-24.3; -3.5)	0.76 (0.62; 0.93)

[1]Patients with a viable pregnancy at ≥12 weeks of gestation

For categorical variables n (%) and exact 95% CI is presented.
For comparison between groups Fisher´s Exact test (2-sided) was used for dichotomous variables.
Confidence Limits calculated using Farrington-Manning.

Table S8 Demographics and Baseline Characteristics - freeze all patients only (FAS Population)

Variable	Study group<br>(n=190)	Control<br>(n=163)	p-value
Maternal age	33.0 (3.5)<br>33 (24; 41)	33.4 (3.6)<br>34 (24; 40)	0.31
Maternal age			
< 25	1 (0.5%)	1 (0.6%)	
25 <= Age < 30	32 (16.8%)	27 (16.6%)	
30 <= Age < 35	98 (51.6%)	73 (44.8%)	
35 <= Age < 40	51 (26.8%)	57 (35.0%)	
40 <= Age	8 (4.2%)	5 (3.1%)	0.47
Paternal age	35.4 (5.2)<br>35 (27; 54)	35.4 (5.6)<br>35 (22; 58)	0.90
Paternal age			
< 25	0 (0.0%)	1 (0.7%)	
25 <= Age < 30	18 (10.7%)	16 (11.7%)	
30 <= Age < 35	59 (34.9%)	51 (37.2%)	
35 <= Age < 40	58 (34.3%)	45 (32.8%)	
40 <= Age	34 (20.1%)	24 (17.5%)	0.39
Reason for infertility (couple)			
No clinical subfertility	12 (6.3%)	9 (5.5%)	
Unexplained infertility	71 (37.4%)	60 (36.8%)	
Know cause for infertility	107 (56.3%)	94 (57.7%)	0.94
Maternal height (cm)	166.1 (7.3)<br>166 (143; 185)	165.1 (7.2)<br>165 (130; 183)	0.23
Maternal weight (kg)	70.4 (15.3)<br>66 (44; 118)	69.9 (14.6)<br>68 (46; 133)	0.73
Maternal BMI	25.5 (5.3)<br>24.3 (16.9; 41.8)	25.6 (5.0)<br>24.5 (18; 43.9)	0.89
Type of menstruation			
Regular cycle	141 (74.2%)	115 (70.6%)	
Irregular cycle	49 (25.8%)	48 (29.4%)	0.47
Number of previous stimulated IVF cycles leading to oocyte pick-up	0.447 (0.870)<br>0 (0; 6)	0.411 (0.822)<br>0 (0; 6)	0.74
Previous pregnancies in current relationship			
0	142 (74.7%)	120 (73.6%)	
1	38 (20.0%)	30 (18.4%)	
2	9 (4.7%)	8 (4.9%)	
>2	1 (0.5%)	5 (3.0%)	0.26

For categorical variables n (%) is presented.
For continuous variables Mean (SD) / Median (Min; Max) / n= is presented.
For comparison between groups Fisher´s Exact test (2-sided) was used for dichotomous variables and the Mantel-Haenszel Chi Square test (2-sided) was used for ordered categorical variables and Chi Square test (2-sided) was used for non-ordered categorical variables and the Fisher´s Non Parametric Permutation Test (2-sided) was used for continuous variables.

Table S9 Pregnancy follow-up (PP population)

Variable	Study group (n=500)	Control (n=502)	p-value
Birth of at least one child, per randomized patient	204/500 (40.8%)	223/502 (44.4%)	0.25
Singletons	199/204 (97.5%)	220/223 (98.7%)	0.49
Twins	5/204 (2.5%)	3/223 (1.3%)	0.49
Male, in singletons	106/198 (53.5%)	129/220 (58.6%)	0.32
Gestational age (weeks), in singletons	39.0 (1.7)<br>39.1 (32.4; 42.4)<br>n=199	38.8 (2.1)<br>39 (23.1; 41.7)<br>n=220	0.28
Very preterm birth (<32 weeks)	0/199 (0.0%)	3/220 (1.4%)	0.25
Preterm birth (<37 weeks)	19/199 (9.5%)	24/220 (10.9%)	0.75
Live birth (>=37 weeks)	180/199 (90.5%)	196/220 (89.1%)	0.75
Birth weight (g), in singletons	3297 (562)<br>3300 (1719; 4800)<br>n=196	3268 (585)<br>3300 (570; 4564)<br>n=217	0.62
Health problems in child 1	14/198 (7.1%)	11/220 (5.0%)	0.41
Health problems in second twin	1/5 (20.0%)	0/3 (0.0%)	1.00
Number of clinical pregnancies	237/500	245/502	
Reason no birth, by fetal heartbeat			0.23
Stillborn child	1/237 (0.4%)	0/245 (0.0%)	
Induced abortions for fetal abnormality	6/237 (2.5%)	2/245 (0.8%)	
Spontaneous miscarriage before week 20	22/237 (9.3%)	19/245 (7.8%)	
Fetal death in utero after 20 weeks	3/237 (1.3%)	0/245 (0.0%)	
Induced abortion other reasons	1/237 (0.4%)	1/245 (0.4%)	
Ectopic pregnancy	0/237 (0.0%)	1/245 (0.4%)	1.00

For categorical variables n/N (%) is presented.
For continuous variables Mean (SD) / Median (Min; Max) / n= is presented.
For comparison between groups Fisher´s Exact test (2-sided) was used for dichotomous variables and Chi Square test (2-sided) was used for non-ordered categorical variables and the Fisher´s Non Parametric Permutation Test (2-sided) was used for continuous variables.

Table S10 Clinical pregnancy by iDAScore agreement groups (iDAScore group, ITT Population)

Variable	The agreement group (n=344)	Doesn't agree with the embryologist (n=179)	p-value	Difference between groups Mean (95% CI)
Clinical pregnancy with fetal heartbeat after the first embryo transfer	166 (48.3%) (42.9%-53.7%)	80 (44.7%) (37.3%-52.3%)	0.46	-3.6 (-13.0; 5.9)
For categorical variables n (%) and exact 95% CI is presented.<br>For comparison between groups Fisher´s Exact test (2-sided) was used for dichotomous variables.<br>10 missing values for the evaluation				

Table S11 Exploratory investigation of the Gardner Grade of the transferred blastocyst in the agreement and non-agreement groups in the iDAScore group(FAS Population)

	iDAScore Group	
Morphology	The agreement group (n=331)	The disagreement group (n=173)
1	0.3% (1)	1.1% (2)
2	2.3% (8)	5.6% (10)
3AA	2.0% (7)	2.2% (4)
3AB	1.2% (4)	1.1% (2)
3AC	0.3% (1)	0.0% (0)
3BA	1.2% (4)	2.8% (5)
3BB	1.7% (6)	2.8% (5)
3BC	0.0% (0)	2.2% (4)
3CB	0.0% (0)	0.6% (1)
3CC	0.0% (0)	1.1% (2)
4AA	36.0% (124)	27.4% (49)
4AB	8.7% (30)	8.4% (15)
4AC	0.0% (0)	0.6% (1)
4BA	4.4% (15)	2.8% (5)
4BB	3.2% (11)	7.8% (14)
4BC	0.9% (3)	0.6% (1)
4CB	0.0% (0)	0.0% (0)
4CC	0.9% (3)	1.2% (2)
5AA	29.6% (98)	16.2% (28)
5AB	3.9% (13)	3.5% (6)
5AC	0.0% (0)	0.6% (1)
5BA	1.2% (4)	4.0% (7)
5BB	0.6% (2)	3.5% (6)
5BC	0.3% (1)	1.2% (2)
5CA	0.0% (0)	0.6% (1)
5CC	0.6% (2)	1.2% (2)
6AA	0.3% (1)	0.0% (0)
6AB	0.0% (0)	0.6% (1)
6BA	0.0% (0)	0.0% (0)
Unknown	0.3% (1)	0.0% (0)

Table S12 Comparison of laboratory outcomes between patients who underwent a fresh transfer and those who underwent a frozen transfer after a freeze-all cycle (FAS population)

	Fresh transfer (n=673)	Freeze-all (n=353)	p-value
Number of oocytes retrieved	11.7 (11.3-12.0)	20.4 (19.3-21.4)	<0.001
Number of fertilized 2PN oocytes	7.3 (7.0-7.5)	12.3 (11.6-13.0)	<0.001
Number of Day 5 blastocysts	4.2 (4.0-4.4)	7.0 (6.6-7.5)	<0.001
Number of cryopreserved embryos[1]	3.2 (3.0-3.4)	6.9 (6.4-7.4)	<0.001

[1]Patients in the fresh transfer had one blastocyst transferred whereas patients in the freeze all had all suitable embryos cryopreserved

All results are presented as mean (95% CIs) and the p-values produced via a Wilcoxon rank sum test (2-sided).

Table S13 Exploratory investigation of the Gardner grade of the transferred embryo in the study and control group. Comparison for all transfers and the fresh and frozen subgroup (FAS Population)

	All			Fresh			Freeze All	
Morphology	Study group (n=510)	Control (n=516)		Study Group (n=320)	Control (n=353)		Study group (n=190)	Control (n=163)
1	3 (0.6%)	4 (0.8%)		1 (0.3%)	1 (0.3%)		2 (1.1%)	3 (1.8%)
2	18 (3.5%)	11 (2.1%)		16 (5.0%)	8 (2.3%)		2 (1.1%)	3 (1.8%)
3AA	11 (2.2%)	9 (1.7%)		8 (2.5%)	9 (2.5%)		3 (1.6%)	0 (0.0%)
3AB	6 (1.2%)	14 (2.7%)		3 (0.9%)	10 (2.8%)		3 (1.6%)	4 (2.5%)
3AC	1 (0.2%)	0 (0.0%)		0 (0.0%)	0 (0.0%)		1 (0.5%)	0 (0.0%)
3BA	9 (1.8%)	6 (1.2%)		7 (2.2%)	5 (1.4%)		2 (1.1%)	1 (0.6%)
3BB	11 (2.2%)	11 (2.1%)		6 (1.9%)	10 (2.8%)		5 (2.6%)	1 (0.6%)
3BC	4 (0.8%)	0 (0.0%)		3 (0.9%)	0 (0.0%)		1 (0.5%)	0 (0.0%)
3CB	1 (0.2%)	1 (0.2%)		1 (0.3%)	0 (0.0%)		0 (0.0%)	1 (0.6%)
3CC	2 (0.4%)	0 (0.0%)		0 (0.0%)	0 (0.0%)		2 (1.1%)	0 (0.0%)
4AA	167 (32.7%)	180 (34.9%)		111 (34.7%)	120 (34.0%)		56 (29.5%)	60 (36.8%)
4AB	41 (8.0%)	32 (6.2%)		26 (8.1%)	28 (7.9%)		15 (7.9%)	4 (2.5%)
4AC	2 (0.4%)	3 (0.6%)		1 (0.3%)	1 (0.3%)		1 (0.5%)	2 (1.2%)
4BA	20 (3.9%)	13 (2.5%)		14 (4.4%)	7 (2.0%)		6 (3.2%)	6 (3.7%)
4BB	25 (4.9%)	18 (3.5%)		17 (5.3%)	13 (3.7%)		8 (4.2%)	5 (3.1%)
4BC	4 (0.8%)	2 (0.4%)		2 (0.6%)	2 (0.6%)		2 (1.1%)	0 (0.0%)
4CB	0 (0.0%)	1 (0.2%)		0 (0.0%)	1 (0.3%)		0 (0.0%)	0 (0.0%)
4CC	5 (1.0%)	4 (0.8%)		4 (1.2%)	4 (1.1%)		1 (0.5%)	0 (0.0%)
5AA	126 (24.7%)	162 (31.4%)		66 (20.6%)	104 (29.5%)		60 (31.6%)	58 (35.6%)
5AB	19 (3.7%)	14 (2.7%)		13 (4.1%)	12 (3.4%)		6 (3.2%)	2 (1.2%)
5AC	1 (0.2%)	0 (0.0%)		1 (0.3%)	0 (0.0%)		0 (0.0%)	0 (0.0%)
5BA	11 (2.2%)	17 (3.3%)		5 (1.6%)	10 (2.8%)		6 (3.2%)	7 (4.3%)
5BB	8 (1.6%)	7 (1.4%)		6 (1.9%)	4 (1.1%)		2 (1.1%)	3 (1.8%)
5BC	3 (0.6%)	1 (0.2%)		1 (0.3%)	1 (0.3%)		2 (1.1%)	0 (0.0%)
5CA	1 (0.2%)	0 (0.0%)		0 (0.0%)	0 (0.0%)		1 (0.5%)	0 (0.0%)
5CC	4 (0.8%)	1 (0.2%)		3 (0.9%)	1 (0.3%)		1 (0.5%)	0 (0.0%)
6AA	1 (0.2%)	2 (0.4%)		1 (0.3%)	0 (0.0%)		0 (0.0%)	2 (1.2%)
6AB	1 (0.2%)	0 (0.0%)		0 (0.0%)	0 (0.0%)		1 (0.5%)	0 (0.0%)
6BA	0 (0.0%)	1 (0.2%)		0 (0.0%)	0 (0.0%)		0 (0.0%)	1 (0.6%)
Unknown	5 (1.0%)	2 (0.4%)		4 (1.2%)	2 (0.6%)		1 (0.5%)	0 (0.0%)

EVALUATING IDA SELECTION ABILITY. THE VISA STUDY.

WILL EMBRYO SELECTION THROUGH USE OF ARTIFICIAL INTELLIGENCE (IDA) PERFORM EQUALLY COMPARED TO BLASTOCYST SCORING?

Study Title/Acronym	
Reference number(s)	
Version	21
Date	4 February, 2022
Sponsor	Vitrolife A/S
Principal Investigator	A/Prof. Peter Illingworth

1 STATEMENTS OF COMPLIANCE

The clinical investigation shall be conducted in accordance with the ethical principles in the Declaration of Helsinki and in compliance with this document and any regional or national regulations.

The clinical investigation will not commence until the required approval/favorable opinion from the ethical committee and regulatory authority have been obtained. Any additional requirements imposed by these institutions shall be followed.
A clinical investigations agreement has been signed between the sponsor and the investigation site(s).

1.1 Approval and agreement

The Principal Investigator and the Sponsor agree to perform this investigation in accordance with this clinical investigation plan (CIP), additional study documentation and any relevant regulatory requirements.

No changes to this clinical investigation plan will be permitted without the approval of both parties. If clinical investigation plan changes become necessary, written approval by the Ethics Committee will be obtained before the changes are implemented.

2 AMENDMENTS TO THE CLINICAL INVESTIGATION PLAN

This Clinical Investigation Plan (CIP) is amended when needed, such as if new information regarding the investigational device is available. If the amendment impacts the integrity of the clinical investigation, data collected before and after the amendment will be statistically analysed and the effect of the amendment on performance, effectiveness or safety analysis will be assessed. This analysis is included in the clinical investigation report.

Proposed amendments to the CIP are reviewed and approved by the same parties as specified on the signature page, unless specifically designated otherwise. The amendments to the CIP shall be notified to, or approved by, the EC and regulatory authorities, as required. The changes are described in the below table, and, where relevant, a justification for and assessment of the potential impact on performance, effectiveness, safety or other endpoints is also documented.

2.1 Revision history

Version	Date published	Amendments to the clinical investigation plan and their justifications
21		The document has been modified to adhere to ISO 14155:2020. This entails both changes in both format and layout. The investigational device is described in greater detail, risks and benefits are highlighted, and the safety reporting procedure has been described.<br><br>Addition of a sub-study, see chapter 15.
20		Submitted to the ethical committee

TABLE OF CONTENTS

1 Statements of compliance 2
1.1 Approval and agreement 2
2 Amendments to the clinical investigation plan 3
2.1 Revision history 3
3 Abbreviations and acronyms 7
4 Contact information 8
5 Overall synopsis of the clinical investigation 9
6 Background and rationale 11
7 The investigational device and the comparator 12
7.1 Identification of the investigational device 12
7.2 Identification of the comparator 13
7.3 Device accountability 13
8 Purpose of the clinical investigation 14
8.1 Hypothesis to be tested 14
8.2 Primary objective 14
8.3 Secondary objectives 14
9 Research design 15
9.1 Primary endpoint 15
9.2 Secondary endpoints 15
9.3 Minimizing bias 15
9.4 Setting 15
9.5 Subjects: 15
9.6 Inclusion criteria 15
9.7 Exclusion criteria 15
9.8 Subject's terms of participation 16
9.9 Criteria and procedures for subject withdrawal or lost to follow-up 16
9.10 Deviations from the clinical investigation plan 16
9.11 Duration of study 16
10 Procedures (IVF) 17
10.1 Subject identification 17
10.2 Informed consent process 17
10.3 Ovarian stimulation 17
10.4 Embryology and randomization-blinding-allocation concealment 17
10.5 Interventions due to study participation 19
10.6 IVF-Cycle outcome 20

10.7 Duration of study ... 21
10.8 Data safety monitoring board (DSMB) ... 21
11 Benefits and risks of the investigational device, clinical procedure, and clinical investigation ... 22
11.1 Anticipated clinical benefits of the investigational device ... 22
11.2 Anticipated adverse device effects in the clinical investigation ... 22
11.3 Risks associated with participation in the clinical investigation ... 22
11.4 Possible interactions with concomitant medical treatments ... 22
11.5 Steps that will be taken to control or mitigate the risks ... 22
11.6 Rationale for benefit-risk ratio ... 23
12 Data collection and management ... 24
12.1 The electronic case form (eCRF) ... 24
12.2 Confidentiality ... 24
12.3 Training ... 24
12.4 Record keeping and archiving ... 24
12.5 Data retention ... 25
13 Monitoring ... 26
13.1 Data safety monitoring board (DSMB) ... 26
14 Statistical design and analysis ... 27
14.1 Definition of study populations ... 27
14.2 Descriptive statistics of baseline data ... 27
14.3 Outcome variables ... 27
14.4 Efficacy and safety variables ... 28
14.5 Sample size calculation ... 28
14.6 General statistical methodology ... 28
14.7 Efficacy analyses ... 29
14.8 Analyses of demographics and treatment variables ... 29
14.9 Exploratory interaction analyses ... 29
15 Substudy within the clinical investigation ... 30
16 Safety evaluation and reporting ... 31
16.1 Definitions ... 31
16.2 Recording and reporting of adverse events ... 31
16.3 Reporting adverse events to all National Competent Authorities ... 32
16.4 Causality assessment ... 33
16.5 Examples of adverse events ... 33
16.6 Summary of registering and reporting events ... 34
16.7 Emergency contacts for reporting SAEs ... 34

17 Publication policy 35
17.1 Main results 35
17.2 Ethical oversight 35
18 References 36
APPENDIX 1. Scoring of day 5-6 embryos 37
Expected Development stages of Embryos-Blastocysts: 37
Blastocyst stage grading: 38

3 ABBREVIATIONS AND ACRONYMS

Abbreviation/ acronym	Definition
AUC	Area under the curve
FSH	Follicle stimulation hormone
GnRH	Gonadotropin-releasing hormone
hCG	Human chorionic gonadotropin
hpi	Hours post insemination
ICSI	Intracytoplasmic sperm injection
iDAScore®	Intelligent data analysis score (for embryo evaluation)
ITT	Intention-to-treat
IVF	in vitro fertilization
MDR	The Medical Device Regulation (EU) 2017/745
PN	Pronucleus
PP	Per protocol
RCT	Randomized controlled trial
ROC	Receiver operating characteristic
SDD	Stockholm Data Design

4 CONTACT INFORMATION

Sponsor	Vitrolife A/S, company reg no. 27406793<br>Postal address: Jens Juls Vej 20, 8260 Viby J, Denmark.
Principal Investigator	A/Prof. Peter Illingworth<br>Medical Director<br>IVFAustralia, 176 Pacific Highway, Greenwich, NSW 2065<br>Peter.illingworth@ivf.com.au
Steering committee	**Vitrolife A/S:**<br>Dr. Thorir Hardarson (thardarson@vitrolife.com)<br>Dr. Mark Larman, Vitrolife AB
	**Virtus Health:**<br>A/Prof. Peter Illingworth<br>Prof. David Gardner<br>Dr. Christos Venetis
	TFP:<br>A/Prof. Scott Nelson
Investigation site(s)	Listed in the List of Investigation Sites
Monitor Arrangements	Vitrolife A/S
Statistical Analysis	Statistiska Konsultgruppen
Data Management	Stockholm Data Design

5 OVERALL SYNOPSIS OF THE CLINICAL INVESTIGATION

Title	**eValuating iDA Selection Ability. The VISA study.** **Will embryo selection through use of artificial intelligence (iDA) perform equally compared to blastocyst scoring?**
**Short title or acronym**	The VISA study
**Clinical registry identifier**	Clinicaltrials.gov: NCT04969822<br>ANZCTR: ACTRN12620000197932
**Type of Investigation**	A randomised controlled multicentre investigation
**Investigational design**	A non-inferiority, prospective parallel group, multi-centered, randomized controlled trial.
**Investigational device**	iDAScore (a software)
**Primary objective**	To investigate whether selection of a single blastocyst for transfer using the deep learning-based support tool called iDAScore results in an equally high clinical pregnancy rate compared to when the selection is performed by trained embryologists using conventional morphology only.
**Secondary objective(s)**	To investigate whether blastocyst selection supported by iDAScore results in equal rates of the below parameters compared to when embryo selection performed by trained embryologists using conventional morphology only:<br>1. Live birth rate<br>2. Positive hCG rate<br>3. Rate of non-viable intrauterine pregnancies<br>4. Ongoing pregnancy rate in patients with maternal age above 35
**Primary endpoint**	Clinical pregnancy as confirmed by an ultrasound and defined as the presence of a fetal heartbeat after 42 days of gestation.
**Secondary endpoints**	1. Live birth<br>2. Positive hCG<br>3. Non-viable intrauterine pregnancy<br>4. Ongoing pregnancy in patients with maternal age >35
**Inclusion criteria**	1. Women undergoing IVF or ICSI with controlled ovarian stimulation with gonadotrophins and the intention to treat by either transfer of a single fresh embryo on day 5 or in case of a freeze all cycle, the first rewarmed embryo.<br>2. Age: Up to and including the 42nd completed birthday on the day of randomization.<br>3. Has at least two early blastocysts on day 5
**Exclusion criteria**	1. Treatment involving donated eggs<br>2. Intention to perform any form of preimplantation genetic testing<br>3. The use of IMSI or polarized light in the ICSI process

	4. The use of assisted hatching to randomization.<br>5. Previous participation in this RCT<br>6. Where the cycle is carried out for fertility preservation.<br>7. If a day 2-4 transfer is planned<br>8. Has a reduced likelihood of obtaining two early blastocysts on day 5 as evidenced by either: AMH level of <3pmol/L or AFC <5 (if available)
Number of participants (rand)	1040
Target population	IVF patients with a planned single blastocyst transfer
Estimated total duration of the investigation	3 years
Estimated duration per participant	1 month – approximately 1 year, depending on outcome
Safety assessments	Safety will be assessed by appropriate recording and reporting of adverse events throughout the investigation as well as by any differences in the endpoints.

6 BACKGROUND AND RATIONALE

Normally, in IVF, embryos are created in the laboratory and cultured for up to six days until they reach the blastocyst stage. On the day of transfer, the embryologist will study the morphologic appearances of the embryos to select the embryo with the highest likelihood of success. The rationale behind which morphological parameters are included vary between clinics as not golden standard exists in choosing the best embryo for transfer. In addition, numerous studies have demonstrated a considerable intra- and inter-observer variability (Paternot *et al*. 2011 and Bendus *et al*. 2006) for cleavage stage embryos and to some extent for the blastocyst stages.

The issue of whether assessment of blastocyst morphology is the optimal method of selecting the embryo with the best chance of developing into a viable pregnancy has been studied extensively over the past twenty years. Several approaches to the study of biomarkers have been evaluated (Simon *et al*., 2015) but, so far, without gaining widespread acceptance. Recently, the advent of additional information from time lapse imaging has been applied to refine the selection criteria with some success (Rubio *et al*., 2014; Goodman *et al*., 2016).

Recently, Virtus Health and Harrison AI developed an Artificial Intelligence (AI) system that studies time lapse images obtained from the embryo culture system, Embryoscope, throughout the development to blastocyst. This technology was subsequently acquired by Vitrolife in April 2019 and has undergone further development. The AI system uses data acquired from a sequence of embryo images and has taught itself to identify the embryos with the highest likelihood of implanting and leading to fetal heart-beat detection. This approach differs from previous algorithms in that it is completely learned and is not dependent on any assumptions from previous knowledge of embryology standards.

The AI system, (iDAScore) has been evaluated through a retrospective analysis of 10,208 embryos with known outcome originating from 1,603 patients between 2014 and 2018 (Tran et al., 2018). This work used ROC curve analysis (Tran *et al*., 2018) to demonstrate that iDA can discriminate between embryos that will or will not result in a pregnancy with a fetal heart with an AUC of 0.93 which appears to be significantly superior to previously published work using existing methods (Adolfsson *et al*., 2018).

The aim of this study is to carry out a randomized controlled trial to investigate whether embryo selection using iDAScore can provide a non-inferior clinical pregnancy rate with fetal heartbeat after transfer of a blastocyst compared to when selection is performed by the laboratory embryologists using the Gardner scoring system (Gardner *et al*. 2000).

7 THE INVESTIGATIONAL DEVICE AND THE COMPARATOR

7.1 Identification of the investigational device

Product name	iDAScore
Software version	1.2
Ref nr	16536
Basic UDI	05712714671005
Manufacturer	Vitrolife A/S<br>Jens Juuls Vej 20, 8260 Viby J, Denmark<br>SRN: DK-MF-000001892
Description	The iDAScore is a software designed to automatically identify embryos with the highest chance of implantation.<br>During embryo culture in an Embryoscope time-lapse system, images of embryo development are captured on multiple focal planes and time intervals and stored on the ES server. Using information from the ES server, iDAScore assigns a score for each embryo based on a deep learning neural network-based algorithm for predicting viability.<br>The device will not come in contact with tissues or body fluids. It does not incorporate any medicinal products or materials of biological origin.<br>The iDAScore was CE marked under the European Medical Devices Directive 93/42/EEC in early 2020.
Risk class	iDAScore is considered a class I accessory for medical devices according to Rule 11 of the Regulation (EU) 2017/745 - Medical Device Regulation.
Intended use	The device evaluates early embryo development through acquired embryo timelapse videos to assist embryo selection. The device assigns a score to each embryo that identifies embryos with the highest chance of implantation. The device is an adjunct to clinical decision-making, and the final assessment and decision must be made by a medical professional.
Indications for use	iDAScore is intended to be used for evaluation of embryos from IVF patients. iDAScore must be used together with an EmbryoScope time-lapse system.
Contraindications	No known contraindications. Contraindications on the patient side regarding a fertility treatment per se are left to the judgement of the physician and are related to the patient. iDAScore does not have any influence on treatment-related contraindications.
Intended target group	There are two different intended target groups. The first group is the patient target group, i.e., patients undergoing fertility treatment. The second group is the user target group, i.e., embryologists, other laboratory personnel and clinic staff at IVF clinics.

Training	User training is included in the installation process. Only trained personnel are to operate the device.
Clinical benefits	As an accessory to a medical device, iDAScore provides the indirect clinical benefit of improving the decision-making process by providing support for selection of embryos incubated in the incubator(s) connected to the system.
Claims (for the medical device, not for the clinical investigation)	- Improved embryo evaluation and selection<br>- Objective embryo evaluation<br>- Consistent embryo evaluation<br>- Automatic embryo evaluation<br>- Reliable embryo evaluation

For more information on the iDAScore, please refer to the Investigator´s Brochure and the User Manual.

7.2 Identification of the comparator

Conventional morphology is used as the comparator in the control group. Trained embryologists will assess the morphologic appearances of the embryos in culture to select the embryo with the highest likelihood of success. Blastocyst stage grading is based on a 3-stage system incorporating the degree of expansion, trophectoderm status, and inner cell mass status (for a detailed description, please see Appendix 1).

Studies have shown conventional morphology to suffer from both intra- and inter-observer variability. Furthermore, also the ranking of embryos, which is based on the scoring of the embryos, may differ between laboratories and individuals. In the current investigation, laboratories are requested to abide to the ranking guideline in Appendix 1.

7.3 Device accountability

N/A.

8 PURPOSE OF THE CLINICAL INVESTIGATION

The purpose of the clinical investigation is to investigate whether embryo selection supported by iDAScore can result in a non-inferior clinical pregnancy rate after blastocyst transfer compared to when selection is performed using the Gardner scoring system.

8.1 Hypothesis to be tested

An embryo selected by the iDA deep-learning system will have a non-inferior chance of clinical pregnancy* after the transfer of the first blastocyst compared to a blastocyst selected by embryologists using conventional morphology.

*Clinical pregnancy is defined in this study as the detection of a fetal heartbeat by ultrasound after 42 days of gestation (week 6).

8.2 Primary objective

To investigate whether selection of a single blastocyst for transfer supported by the deep learning tool, (iDAScore), results in an equally as high clinical pregnancy rate compared to trained embryologists using standard morphology criteria.

8.3 Secondary objectives

1. Live birth rate
2. Positive hCG rate
3. Rate of non-viable intrauterine pregnancies
4. Ongoing pregnancy rate in patients with maternal age above 35

9 RESEARCH DESIGN

A non-inferiority, prospective parallel group, multi-centered, randomized controlled trial.

9.1 Primary endpoint

Clinical pregnancy, defined as the detection of a fetal heartbeat by ultrasound after 42 days of gestation.

9.2 Secondary endpoints

- Live birth
- Positive hCG, determined by a hCG measurement from a blood sample or using urinary sticks
- Non-viable intrauterine pregnancy, as witnessed by miscarriage
- Ongoing pregnancy rate in patients with maternal age above 35

See section 10.6 and chapter 14 for more details.

9.3 Minimizing bias

All embryos are assessed morphologically (“control treatment”) and a preliminary decision on choice of embryo to transfer is made prior to randomization. This is done to ensure the patient meets the inclusion criteria of having a minimum of two early blastocysts, but also to reduce bias.

Both the treating clinician and the patient will remain blinded to the randomization outcome until after the first embryo transfer has been completed. The patient can be told the number of embryos available for transfer and, where clinically indicated, the morphologic grading of each embryo.

Potential confounding factors are considered both in the randomization process through stratification and in the statistical analyses (baseline characteristics, see chapter 14).

9.4 Setting

IVF units performing IVF/ICSI cycles and having Embryoscope and iDAScore capacity.

9.5 Subjects:

IVF patients meeting the criteria of this study and having provided written informed consent.

9.6 Inclusion criteria

1. Women undergoing IVF or ICSI with controlled ovarian stimulation with gonadotrophins and the intention to treat by either transfer of a single fresh embryo on day 5 or in case of a freeze all cycle, the first rewarmed embryo.
2. Age: Up to and including the 42nd completed birthday on the day of randomization.
3. Has at least two early blastocysts on day 5

9.7 Exclusion criteria

1. Treatment involving donated eggs
2. Intention to perform any form of preimplantation genetic testing

3. The use of IMSI or polarized light in the ICSI process
4. The use of assisted hatching to randomization.
5. Previous participation in this RCT
6. Where the cycle is carried out for fertility preservation.
7. If a day 2-4 transfer is planned
8. Has a reduced likelihood of obtaining two early blastocysts on day 5 as evidenced by either: AMH level of <3pmol/L or AFC <5 (if available)

9.8 Subject's terms of participation

Study participation does not entail any additional costs, and, therefore, subjects are not compensated for their participation in the project. Insurance is provided for subjects as required by regulations. Compensation for any injury caused by taking part in this study will be in accordance with the guidelines of the Association of the British Pharmaceutical Industry (ABPI). This applies in cases where it is likely that the injury results from a procedure carried out in accordance with the protocol for the study.

9.9 Criteria and procedures for subject withdrawal or lost to follow-up

The patients are under no obligation to enter the investigation and they can withdraw at any time, without having to give a reason. If a participant, who has given consent, loses capability to consent during the investigation, the participant and all identifiable data will be withdrawn from the investigation. Data which is not identifiable to the research team may be retained.

Reasonable efforts will be made to follow-up outcomes of all participants (ex. telephone, e-mail, mail). Randomised patients will not be replaced if they are lost to follow-up and already registered data will be included in the data analysis. To protect against possible lost to follow-up the total number of randomised patients has been increased by 5%.

9.10 Deviations from the clinical investigation plan

The investigator is not allowed to deviate from the CIP, except to protect the rights, safety and well-being of study participants under emergency circumstances. In these cases, the investigator may proceed without prior approval by the sponsor and the ethical committee. However, they must be documented and reported to the sponsor and the ethics committee as soon as possible.

Any CIP deviations are recorded, reported, and analyzed within the eCRF as soon as possible. Corrective and/or preventive actions resulting from the analysis will be arranged without any delay. Serious protocol breaches, if caused by negligence, willful misconduct or risk patient safety, may lead to the disqualification of an investigator or the principal investigator.

9.11 Duration of study

The estimated patient enrollment period is March 2020 to September 2022. Each subject's participation lasts maximally until the confirmation of a live birth. The completion of a clinical investigation coincides with the last visit of the last subject and when follow-up is complete for the clinical investigation. The data collection is expected to be completed in September 2023. The estimated end date of the clinical investigation (database lock) is October 2023. The clinical investigation may be suspended or prematurely terminated for medical reasons or if advised to so by the DSMB.

10 PROCEDURES (IVF)

10.1 Subject identification

Potentially eligible participants will be identified by staff members from patients undergoing IVF/ICSI treatments at the investigational site(s). Vulnerable subjects/groups will not be approached.

10.2 Informed consent process

Potentially eligible participants will receive information both orally and in writing regarding the investigation, its aim, the methods used and the implications of participation. The information is given by the recruiting physician, a research nurse or an embryologist, after which the patients are given at least 24 hours is to consider their participation. Interested participants will be given an opportunity to ask questions about the study and these will be answered prior to enrolment. Those expressing interest in participating are asked to sign an informed consent form. Patients who give consent are enrolled in the investigation and registered in the electronic study database (for couples undergoing IVF treatment, both individuals need to sign informed consent). Patients will be asked to sign the consent form prior to the oocyte pick-up. No clinical investigation procedures will be conducted prior to taking consent from the participant. The original signed form will be retained at the study site and a copy is given to the participants.

Once written informed consent has been obtained, an electronic case report form (eCRF) is completed to document adherence to the inclusion and exclusion criteria. If a subject fails to fulfil any of these criteria, this will be documented and the signed consent form and completed inclusion/exclusion criteria are kept by the investigator. Any subject not fulfilling the enrolment criteria will not be advanced any further into the clinical investigation.

If any new relevant information regarding the investigational device becomes available during the investigation, the participants will be informed by the research team.

10.3 Ovarian stimulation

Ovarian stimulation will be performed with gonadotrophins (recombinant or urinary) as per routine protocol. The treating physician can decide on the starting dose of gonadotrophins and subsequent adjustments based on clinical judgment. Triggering of final oocyte maturation and oocyte retrieval will be performed as per protocol at each clinic.

10.4 Embryology and randomization-blinding-allocation concealment

Once the oocytes have been retrieved, all embryos will be fertilized by the method of IVF or ICSI as per the individual clinical decision-making and will be incubated in the Embryoscope system until day 5 or 6. As per normal embryoscope protocol, IVF-fertilized embryos will normally be placed in the embryoscope on day 1 and ICSI-fertilized embryos will be placed in the embryoscope on day 0. Exceptions to this can be made due to logistical reasons.

All embryos will be incubated using the Embryoscope time-lapse system. Embryo glue will be used as a transfer media in every case.

No analysis of the embryo will be performed by iDAScore until randomization has occurred and no other time lapse algorithms will be applied to the embryos of patients who have been recruited to this study. After randomization, iDAScore will only be applied to the treatment group.

On day 5 an embryologist will score all embryos according to the Gardner scale by visual selection using the Embryoscope software and make a preliminary decision on which embryo is prioritized for embryo transfer. If two or more early blastocysts are available (i.e. at the developmental stage of 2 or more according to the Gardner scale) between 114 and 118 hours post insemination (hpi), the patient is randomized using a 1:1 ratio. If there are fewer than two early blastocysts, randomization will not take place, and this will be recorded in the eCRF.

If randomized to the control group, the preliminary decision on embryo prioritization for transfer remains unchanged. If, however, the patient is randomized into the treatment group, the sequences of all normally fertilized embryos will be analyzed using the iDAScore software and the embryo with the highest score is prioritized for transfer.

Randomization will be performed with the use of a randomization module within the eCFR program. If, for some reason, randomization from within the eCRF is not possible, manual randomization will be performed by flipping a coin and the randomization outcome shall be registered once the database is accessible.

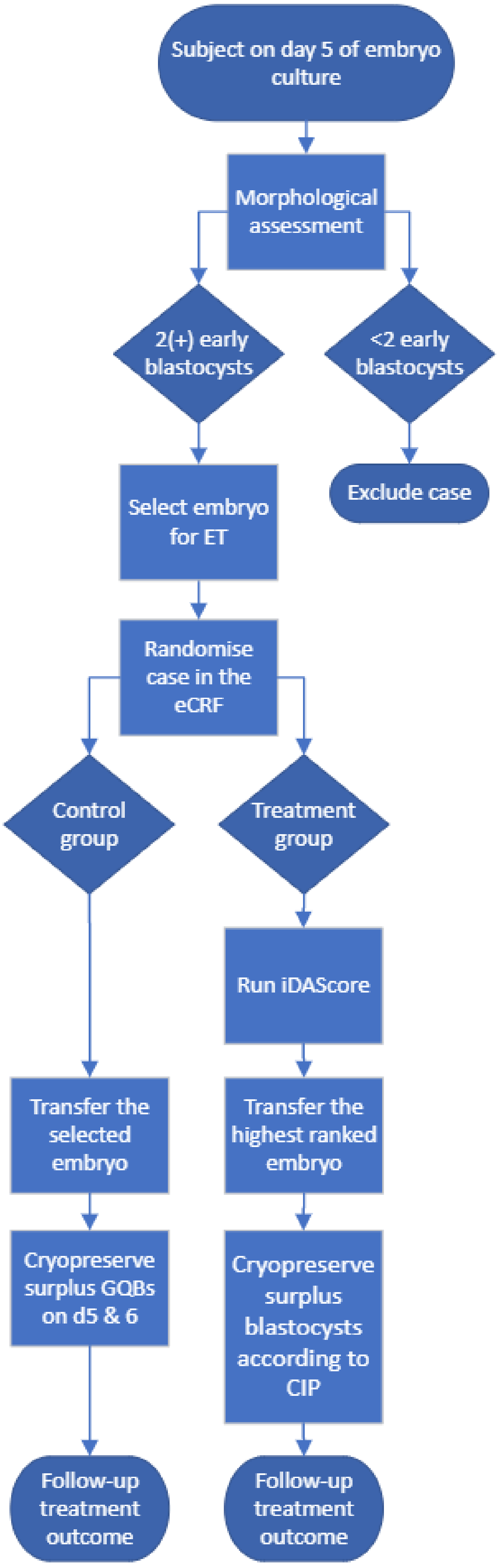

Patients will be randomly allocated to two different study groups:

A. Control group: Embryo selection by conventional morphologic criteria.

- The embryo for transfer will be selected by the embryologist on the basis of the morphologic appearances on day 5, according to the Gardner criteria (Gardner *et al.*, 2000) using the ranking guideline (Appendix 1).

- Regardless of whether a transfer takes place any embryos that fulfil the normal criteria applied in the laboratory for cryopreservation will be cryopreserved (Appendix 1). If there is doubt about whether an embryo is suitable for freezing, embryos may be held over to day 6 and decisions will be made then.

- The prioritization of frozen embryos for later transfer will be made according to Appendix 1 on the basis of the all the information that is available by day 6. Embryos will be warmed in order of this prioritization.

B. Treatment group: Embryo selection supported by iDAScore

- The time-lapse videos will be analyzed by iDAScore at 114-118 hpi and the embryo for fresh transfer on day 5 will be prioritized on the basis of the embryo with the highest iDAScore.

- Any remaining embryos in this group will be cryopreserved that have either:
    - o reached Gardner Grade 3 or beyond AND would normally be frozen or;
    - o have reached Gardner Grade 3 AND achieve a score on iDA of 5 or more.

- All other embryos will be kept to day 6, re-scored at 138-142 hours hpi and reviewed according to the above criteria.

- The warming of embryos will proceed on the prioritization of the iDAScore across the two days. The first embryo to be warmed will be the one with the highest iDAScore. If this embryo does not survive warming and is not suitable for transfer, the next embryo to be warmed will be selected based on the iDAScore, until an embryo is warmed and is suitable for transfer.

Embryo transfer will be performed using the clinic's routine methods. Following transfer of the embryo, luteal support will be administered using the standard protocol of each clinic.

Both the treating clinician and the patient will remain blinded to the randomization outcome until after the first embryo transfer has been completed. The patient can be told the number of embryos available for transfer and, where clinically indicated, the morphologic grading of each embryo.

10.5 Interventions due to study participation

Enrollment in the investigation does not entail any additional testing or extra visits to clinic for the patient. The IVF treatment (ovarian stimulation and embryo culture) is performed according to the clinic's standard operating procedures, except for the method of selecting the embryo for transfer. The randomization result determines the selection method (group affiliation).

Research protocol interventions or procedures

Process	Intervention or procedure	Procedure type	Conducted by clinic staff unless described differently
Work-up prior to patient's IVF treatment	Patient consultation visits prior to treatment start	Routine	
	Medical examination of patient (e.g., ultrasound, blood sampling, sperm analysis)	Routine	
Subject identification	Patient screening according to inclusion/exclusion criteria	Research	
Informed consent process	Informing the patient about the investigation	Research	At the clinic, by telephone or online
	Seeking consent	Research	
Ovarian stimulation	Hormone stimulation treament	Routine	Self-injection, monitoring at the clinic
Embryology	Oocyte pick-up	Routine	
	Sperm analysis and preparation	Routine	
	Fertilisation of oocytes	Routine	
	Embryo culture	Routine	
	Randomisation, allocation, concealment	Research	
	Embryo assessment and selection	Research	
	Embryo transfer	Routine	
	Cryopreservation of surplus embryos	Routine	
	In case of freeze-all cycle, warming of embryos	Routine	
IVF treatment outcome	Pregnancy test	Routine	By the subject at home. If a blood test is required, this is taken at the clinic
	Pregnancy ultrasound (if positive pregnancy test)	Routine	At clinic or maternity center
	Cycle outcome report	Routine	By the subject to the clinic

10.6 IVF-Cycle outcome

The outcome of the cycle will be determined by the following assessments:

- Pregnancy will be tested either through a hCG measurement carried out from Day 9-13 following embryo transfer or using urinary sticks (25 IU/L) on day 13.

- A transvaginal ultrasound performed between 28 and 42 days after the embryo transfer (between 7 and 9 weeks of gestation).

Additional hCG measurements and ultrasounds may be performed according to the directions of the supervising physician.

Based on these assessments, the initial outcome of the first embryo transfer cycle will be categorized as:

**Not pregnant**	Negative test result; urinary hCG<25 IU/L with urine sticks, hCG in blood <50 IU/L
**Biochemical pregnancy**	Positive test result with regard to the above cut-off values, but no other clinical evidence of pregnancy, includes pregnancy of unknown location
**Ectopic pregnancy**	No intrauterine pregnancy and, either ultrasound evidence of a fetal heart outside the uterus or histopathological evidence of ectopic pregnancy excised by laparoscopy.
**Non-viable intrauterine pregnancy**	Ultrasound evidence of an intrauterine pregnancy but with no fetal heart observed after 7-9 weeks of gestation.
**Clinical pregnancy with fetal heartbeat**	Ultrasound evidence of an intrauterine pregnancy with a fetal heart observed after 7-9 weeks of gestation.

In the case of a clinical pregnancy with fetal heartbeat, the eventual outcome of the pregnancy will be followed and recorded.

10.7 Duration of study

Patient recruitment is planned between March 2020 to September 2022. Data collection will proceed until September 2023 (for live birth follow-up).

10.8 Data safety monitoring board (DSMB)

An independent DSMB will be appointed to follow the safety and efficacy monitoring as well as the overall conduct of the study. The board will consist of a statistician and a medically knowledgeable person unrelated to the study. The role of the DSMB will be set out in a separate charter and the members will hold regular meetings where the study efficacy and safety will be assessed and if necessary suggestions for changes in study protocol. For early termination of efficacy for benefit (clinical pregnancy substantially higher in iDA group than in the trained embryologist group) the DSMB should use O'Brian-Fleming's sequential boundaries on the positive side. The DSMB should start to look at efficacy data for benefit after 50% of the subjects have completed evaluation of the primary outcome.

For early termination for harm (clinical pregnancy substantially lower in iDA group than in the trained embryologist group) the DSMB should use a Z value of -2.4 and perform first analysis when 20% of subjects have completed evaluation of the primary outcome. All analyses performed by the DSMB will be strict blinded for everybody outside the DSMB.

11 BENEFITS AND RISKS OF THE INVESTIGATIONAL DEVICE, CLINICAL PROCEDURE, AND CLINICAL INVESTIGATION

11.1 Anticipated clinical benefits of the investigational device

If embryo selection supported by deep learning is superior to the conventional selection technique, the time to pregnancy may be shorter in the treatment group. Occasionally, some embryos in the treatment group may not fulfil the conventional criteria of cryopreservation but reach an acceptance level iDAScore®. These embryos will also be cryopreserved. This means that in some treatment cycles some extra embryos will be cryopreserved that otherwise would have been discarded. This will, if anything, increase the chance of a live birth as these embryos will be transferred later.

11.2 Anticipated adverse device effects in the clinical investigation

The identified risks for the investigational device are described in the investigator's brochure. The table below lists risks anticipated adverse device effects in the current clinical investigation.

Problem	Affects	Caused by	Subject withdrawn	Solution
No iDAScore calculated	Treatment group	Technical issues	Yes	Conventional morphology is used to select an embryo for transfer.
Highest scoring embryo overruled	Treatment group	iDAScore ranking	Yes	Conventional morphology is used to select an embryo for transfer.
No pictures of embryos	Both groups	Technical issues	Yes	Culture dish is removed from the incubator and conventional morphology is assessed using an external microscope.

11.3 Risks associated with participation in the clinical investigation

If embryo selection supported by iDAScore is inferior to the conventional selection technique, subsequent embryo transfers may be required to achieve a pregnancy. This means that the time to pregnancy may be longer for the participants in the treatment group as an embryo with lower potential would have been selected prior to one with higher potential. However, the total chance of becoming pregnant during the IVF treatment is not negatively affected by participating as all surplus embryos of good quality are cryopreserved for later use.

11.4 Possible interactions with concomitant medical treatments

Not applicable.

11.5 Steps that will be taken to control or mitigate the risks

All participants are patients undergoing IVF treatments at the participating study sites, who have signed an informed consent form and want to participate in the clinical investigation. They have been informed about the potential risks and benefits, acknowledge that their participation is voluntary, are aware that they can withdraw at any time. Only patients with a minimum of two early blastocysts (not all available embryos) are included in the study.

This is to ensure minimum quality of morphology and development stage of the embryo to be transferred.

If a blastocyst of lower potential in transferred in the treatment group, the time to pregnancy may be prolonged. However, surplus embryos are cryopreserved for later use. The side-effects of repeated transfer are significantly lower than the effects of a new stimulation cycle.

Laboratories are recommended to double-check the embryo images daily to ensure sufficient quality for iDAScore calculation. The Embryoscope incubator and its software is regularly serviced to ensure proper function.

11.6 Rationale for benefit-risk ratio

The iDAScore is a non-invasive medical device with a very low risk of harm. Previous retrospective studies have shown iDAScore to be able to rank embryos according to their implantation potential. The possible benefits are assessed to outweigh the risks.

12 DATA COLLECTION AND MANAGEMENT

Data from the study will be collected using a dedicated, secure on-line database through the services of Stockholm data design (SDD). The server is placed in Stockholm, Sweden. The randomization software is embedded in the database.

12.1 The electronic case form (eCRF)

Each enrolled subject will be given a unique identifying number in the eCRF. The participation is also be registered in the medical records at the investigational site.

After giving consent to participate in the investigation, information necessary to conduct the study will be recorded in accordance with the patient consent form, the patient information sheet and this clinical investigation plan. Each participant is allocated a unique trial number. This data will be collected on an electronic case report form (eCRF), on a secure server. The clinic will be responsible for completion of an eCRF for each participant. The eCRF will include participant details (initials and unique trial number), medical history, information about the treatment and its outcome, any related adverse events and details of withdrawal from the study if appropriate. The study database will not contain any direct identifiable data such as names or ID numbers.

12.2 Confidentiality

Technical, administrative and physical measures are used to protect personal data from being accessed, disclosed, altered or destroyed by unauthorized persons. These measures include, but are not limited to, individual timestamp login, individual user-ID, two factor authentication access, and encrypted data communication.

All data will be handled in accordance with regulations. The participant's initials, date of birth and trial identification number, will be used for identification, which is explained in the Patient information sheet. Study data will be kept confidential and managed in accordance with applicable regulations, and Research Ethics Committee Approval. No study reports will contain identifiable information. The study data will be anonymised in the clean file.

12.3 Training

Before the investigation is commenced, the sponsor will ensure the investigators and the site staff comprehend the purpose and procedures of the clinical investigation as well as are trained on the investigational medical device and using the eCRF. Hereafter, the investigator is responsible for ensuring that the staff at the investigation site follow the study protocol. All training is documented in a Site Training Log. Key documents are:

- Clinical Investigation Plan (CIP)
- Investigators Brochure (IB)
- The informed consent forms and patient information sheet
- electronic Case Report Forms (eCRFs)
- Instructions For Use (IFUs) or User Manuals (UMs)
- All written clinical investigation agreements, as appropriate

12.4 Record keeping and archiving

All essential documentation will be archived securely by the investigators for a minimum of 10 years after the completion, termination or discontinuation of the investigation. Essential

documents are those which enable both the conduct of the trial and the quality of the data produced to be evaluated and show whether the site complied with all applicable regulatory requirements. The sponsor will notify the study sites when trial documentation can be archived. All archived documents must continue to be available for inspection by appropriate authorities upon request. If the investigator retires, relocates, or for other reasons withdraws from the responsibility of keeping the study records, custody must be transferred to a person who will accept the responsibility

12.5 Data retention

Personal data is registered in the study database in a pseudonymized form. Within 12 months following the completion of the investigation, the data in the study database will be modified and anonymized.

13 MONITORING

Study monitoring activities include study initiation meetings, quarterly remote monitoring and close-out meetings. The study initiation meeting enables the study monitor and/or sponsor to review thoroughly the study protocol and case report forms with the investigator's staff. The degree of monitoring will be proportionate to the risks associated with the investigation. The investigation has been classified as low risk.

The monitor will review case report forms to ensure the completeness and consistency of collected data. The subjects' clinical records will be reviewed to confirm that the case report form data is consistent with the clinical records and to determine whether recording of adverse events has been omitted in the case report forms. The site's Study Regulatory binder and other study documents will be reviewed.

13.1 Data safety monitoring board (DSMB)

An independent DSMB will be appointed to follow the safety and efficacy monitoring as well as the overall conduct of the study. The board will consist of a statistician and a medically knowledgeable person unrelated to the study. The role of the DSMB will be set out in a separate charter and the members will hold regular meetings where the study efficacy and safety will be assessed and if necessary, suggestions for changes in study protocol. The DSMB should start to look at efficacy data for benefit after 50% of the subjects have completed evaluation of the primary outcome.

For early termination for harm (substantially lower clinical pregnancy rate in the iDAScore® group than in the control group) the DSMB should use a Pocock's sequential boundaries on the negative side and perform first analysis when 20% of subjects have completed evaluation of the primary outcome. All interim analyses will be performed by the DSMB and will be strict blinded for everybody outside DSMB.

14 STATISTICAL DESIGN AND ANALYSIS

A statistical analysis plan (SAP) will include a detailed description of all statistical analyses. Data for statistical analyses will be provided by creating a clean file from the study database. The clean file will then be anonymized before any statistical analyses will be performed.

14.1 Definition of study populations

Population	Definition
Intention-to-Treat (ITT) population	All randomized subjects.
Full Analysis Set (FAS)	All randomized subjects with measurement of primary efficacy variable.
Per protocol (PP) population	All randomized subjects without significant protocol violations.
Safety population	All enrolled subjects.

The final decisions regarding all the above study populations will be taken at the Clean-File-meeting before the database lock.

14.2 Descriptive statistics of baseline data

Main demographic parameters (Demographics and baseline characteristics)	Main treatment parameters (Treatment variables)
1. Age (maternal*/paternal)<br>2. Reason for infertility (couple)<br>3. Hight and weight (mat.)<br>4. BMI (mat.)<br>5. Type of menstruation (mat.)<br>6. Number of previous stimulated IVF cycles leading to oocyte pick-up (couple)*<br>7. Previous pregnancies in current relationship<br>8. Previous appearance of a FH at week 6-9, in current relationship<br>9. Previous births, after week 20 of gestation, in current relationship<br><br>*) These parameters are used for stratification during randomisation	1. FSH brand and starting dosage<br>2. FSH total dosage<br>3. GnRH downregulation (agonist/ antagonist)<br>4. Source of sperm<br>5. Duration of ovarian stimulation<br>6. Number of oocytes*<br>7. Method of fertilization (ICSI/Standard IVF/Combined)*<br>8. Number of normally fertilized oocytes (2PN)*<br>9. Number of embryos available for selection<br>10. Number of cryopreserved embryos on day 5 and 6<br>11. Morphological score of the transferred embryo<br>12. iDAScore® (treatment group) of the transferred embryo<br>13. Did AI change the decision of the embryologist? (Y/N)<br>14. Type and duration of LH support

14.3 Outcome variables

1. Biochemical Pregnancy (Y/N)

2. Clinical pregnancy (Y/N)
3. Number of sacs
4. Live birth (Y/N)
5. Reason for pregnancy loss
6. Birth weight (if applicable)
7. Child gender

14.4 Efficacy and safety variables

In the investigation, the efficacy variables are also considered to be safety variables as a possible negative effect of the iDAScore could be reflected in the pregnancy outcomes.

Primary efficacy variable

Rate of clinical pregnancy with fetal heartbeat after the first embryo transfer cycle between the control and treatment groups (comparing embryo selection by standard morphologic criteria with embryo selection by iDAScore).

Secondary efficacy variables

- Live birth rate
- Positive hCG rate per randomized patient
- Rate of non-viable intrauterine pregnancies (as described above)
- Ongoing pregnancy rate in patients with maternal age above 35

14.5 Sample size calculation

It is estimated from the results in clinics that clinical pregnancy is estimated to be 35.4% for trained embryologists. If non-inferiority margin is defined as - 5%, the lower limit of the two-sided 95% confidence interval (CI) for the difference between iDAScore group and Trained embryologist group shall not be less than -5% with a probability of 90% (β=10%), with an estimation of 5% or more clinical pregnancies in iDAScore group, 494 women per randomization group is needed to show non- inferiority. For protection against a 5% loss to follow-up, 1040 patients in total, 520 per group, are needed for recruitment.

14.6 General statistical methodology

The main statistical analyses will be calculation of the mean percentage difference with 95% confidence interval (CI) regarding pregnancy rate between the iDAScore group and the trained embryologist group. For comparisons between the two randomized groups Fisher's exact test will be used for dichotomous variables, Fisher's non-parametric permutation test for continuous variables, Mantel-Haenszel chi-square test for ordered categorical variables and Pearson chi-square test for non-ordered categorical variables.
For dichotomous variables mean differences and relative risk with 95% CI will be calculated. For continuous variables mean differences between the two groups with 95% CI will be calculated based on Fisher's permutation test and Effect size will be calculated.

Dichotomous data will be expressed as numbers and percentages. Continuous variables will be described with mean, standard deviation, median, quartile 25% and quartile 75%.
If baseline confounders, variables that differ between the randomized groups and predict outcome variables, are found then complementary analyses will be performed adjusted for these baseline variables. For adjusted analyses, multivariable logistic regression will be used for dichotomous outcome variables and ANCOVA for continuous outcome variables.

All primary and secondary analyses will be performed on both the ITT-population and the PP population.
All significance tests will be two-sided and conducted at the 5% significance level.

14.7 Efficacy analyses

The primary statistical analysis will be calculation of the mean percentage difference with 95% confidence interval (CI) regarding pregnancy rate between the iDA group and the trained embryologist group. If the lower limit of this 95% CI exceeds -5%, the non-inferiority margin, then non-inferiority is achieved. If non-inferiority is achieved, then we could test for superiority which means that the lower limit of the above 95% CI will be greater than zero. This analysis will be applied on the PP population (primary) and ITT population (sensitivity analysis). Any inconsistencies in these analyses will be discussed in the study report.

The primary analysis will also be performed adjusted by center and performed per center. If baseline confounders are found the adjusted analyses will be performed adjusted for these variables.

The secondary efficacy analyses will be the comparison between the two randomized group regarding the secondary outcome variables given in section secondary outcomes.

14.8 Analyses of demographics and treatment variables

Demographics and treatment variables will be summarized by randomization groups according to the principles in general statistical methodology above.

Subgroup analysis will be performed on the primary and important secondary variables for the following baseline subgroup:
Maternal age >35

14.9 Exploratory interaction analyses

Exploratory interaction analyses between the two randomized group for the following baseline and primary efficacy variable:

- FSH total dosage
- Number of normally fertilized oocytes (2PN)
- Number of embryos available for selection

If interaction p-value <0.10 then subgroups analysis will follow.

15 SUBSTUDY WITHIN THE CLINICAL INVESTIGATION

Title of substudy: Comparing time used for embryo evaluation between the conventional morphology group (control) and iDAScore (treatment) group.

Background: One of the potential benefits of using artificial intelligence for embryo selection is time saving given that iDAScore will be at least as reliable in ranking embryos as morphology alone. Therefore, we intend to compare the time used to select an embryo for transfer using the two selection methods in the VISA study (i.e. morphology and iDAScore). The null hypothesis is that there will be no differences between the two methods.

Materials and methods: The SD for the difference between the morphological and iDAScore assessment was estimated as 60.6 seconds from a pilot study of 20 patients. To find a mean difference in times between the morphological and iDAScore assessment of 45 seconds with a two-sided Fisher's non-parametric permutation test for paired observations, on significance level 0.05, with a power of 80% then a minimum of 51 patients are needed.

This sub-study will be performed in at least 2 laboratories. To make sure we include cohorts of embryos ranging from few to many, the sites will score embryos of three categories small (2-5 embryos), medium (6-10) and large (>10).

Time for embryo evaluation will measured in the following way:
Once a patient file has been opened in the embryo viewer of the Embryoscope a timer will be started, and embryo scoring performed on all normally fertilized embryos (2PN) using Gardner score. The timer will be stopped once an embryo for transfer has been selected. If the patient is allocated to the treatment group, the timer will be started once a patient file has been opened. Once the iDAScore program has rendered the ranking and an embryo identified with the highest iDAScore the timer will be stopped.

In each of the sites we will analyse the time difference with Fisher's non-parametric permutation test for paired observations and make estimates of the differences between the morphological and iDAScore assessments. The means with 95% CI, SD, median, minimum, and maximum will be calculated.

16 SAFETY EVALUATION AND REPORTING

Deviations from SOPs or the CIP as well as adverse device effects, device deficiencies and adverse events occurring in the context of the investigation are documented. The definitions and reporting requirements adopted in this investigation are derived from the international standard on clinical investigations ISO 14155:2020, the Medical Device Regulation 2017/745 (MDR), MEDDEV 2.7/3. and MDCG 2020-10/1.

16.1 Definitions

Adverse Event (AE)	Any untoward medical occurrence, unintended disease or injury, or any untoward clinical signs (including abnormal laboratory findings) in the context of a clinical investigation in a subject, user, or other person. For users or other persons, this definition is restricted to events related to the use of investigational medical devices or comparators.<br>An adverse device effect (ADE) is a subtype of adverse events. These are adverse events related to the use of an investigational medical device or the comparator (if it is a medical device) and include:<br>1. any adverse event resulting from insufficient or inadequate instructions for use, deployment, implantation, installation or operation, or any malfunction of the investigational medical device.<br>2. any event resulting from use error or intentional misuse of the investigational medical device.
Serious Adverse Event (SAE)	Any adverse event that led to:<br>• death<br>• serious deterioration in health of the subject, user, or other person:<br>1. a life-threatening illness or injury<br>2. a permanent impairment of a body structure or function, including chronic diseases<br>3. in-patient or prolonged hospitalization<br>4. medical or surgical intervention to prevent life threatening illness, injury or permanent impairment to a body structure or function<br>• fetal distress, fetal death, a congenital abnormality, or birth defect.<br>Planned hospitalization for pre-existing condition, or a procedure required by the CIP, without a serious deterioration in health, is not a serious adverse event.<br>A serious adverse device effect (SADE) is a subtype of serious adverse events. These are serious adverse events related to the use of an investigational medical device or the comparator (if it is a medical device).
Device deficiency	Any inadequacy of the investigational device or the comparator in the identity, quality, durability, reliability, usability, safety, or performance. This includes malfunctions, use errors and inadequate supply of information.

16.2 Recording and reporting of adverse events

The following events are registered (MDR 80:1):

- any adverse event of a type identified in the clinical investigation plan as being critical to the evaluation of the results of that clinical investigation
- any serious adverse event

- any device deficiency that might have led to a serious adverse event if appropriate action had not been taken, intervention had not occurred, or circumstances had been less fortunate
- any new findings in relation to the above events

The events will be registered by the clinic within the adverse events module in the eCRF in a timely manner.
All AEs will be characterized by the following criteria:

- Date of incident
- Intensity or Severity
- Relationship to the investigational device and the related procedure
- Outcome
- Treatment or Action Taken

The investigator shall report events **immediately, but not later than 3 calendar days** after investigational site study personnel's awareness of the event. Any serious adverse events during treatment will be reported as required by national regulations to the relevant authorities. The sponsor is responsible for reviewing all adverse events and ensuring they, if necessary, are reported to the EC and regulatory authorities. The patients involved should be informed as soon as possible. Furthermore, they should be offered counselling and/or support.

If unforeseen technical issues occur, regardless of group affiliation, embryos for transfer will be selected based on morphological assessment only.

16.3 Reporting adverse events to all National Competent Authorities

The following are reported by the sponsor:
- any **serious adverse event (SAE)** having a causal or reasonably possible causal relationship with the
  a) investigational device
  b) comparator
  c) investigation procedure
- any **Device Deficiency that might have led to a SAE** if:
  a) appropriate action had not been taken or
  b) intervention had not been made or
  c) if circumstances had been less fortunate
- **new findings/updates** in already reported events.

All reportable events regarding an imminent risk of death, serious injury, or serious illness and that requires prompt remedial action for other patients/subjects, users or other persons are reported **immediately, but not later than 2 calendar days** after awareness by the sponsor. The same deadline applies for updates/new findings regarding this type of events.

Any SAEs reported via the eCRF will also be handled under the post-market surveillance/vigilance system.

Any other reportable events are reported **immediately, but not later than 7 calendar days** after awareness by the sponsor. The same deadline applies for updates/new findings regarding this type of events.

16.4 Causality assessment

The relationship between the use of the medical device and the occurrence of each adverse event shall be assessed and categorized. During causality assessment, clinical judgement shall be used and relevant documents (the Investigator's Brochure, the Clinical Investigation Plan or the Risk Analysis Report) shall be consulted. The presence of confounding factors, such as concomitant medication/treatment, the natural history of the underlying disease, other concurrent illness or risk factors shall also be considered. These considerations apply to all serious adverse events, regardless of whether the subject of the event belongs to the control or the investigational group.

Serious adverse events related to the investigational device will be distinguished from those related to the procedures (any procedure specific to the clinical investigation). An adverse event can be related both to procedures and the investigational device. Complications of procedures are considered not related if the procedures would have been applied to the patients also in the absence of investigational device use/application.

Each SAE will be classified according to five levels of causality. The following definitions are used:

Classification of causality	A relationship between the SAE and the investigational device or procedures…
Not related	can be excluded
Unlikely	seems not relevant and/or the event can be reasonably explained by another cause
Possible	is weak but cannot be ruled out completely.<br>Cases where relatedness cannot be assessed, or no information has been obtained should also be classified as possible.
Probable	seems relevant and/or the event cannot reasonably be explained by another cause.
Causal relationship confirmed	is beyond reasonable doubt.

16.5 Examples of adverse events

Adverse events (AEs) can occur within any clinical or laboratory process (oocyte pick-up, insemination, handling, embryo transfer, cryopreservation) and for instance be caused by the human factor, absence/failure of witnessing or poor-quality systems. The consequences may include reduced/no chance of pregnancy, transmission of disease, psychological impact, and ethical/legal issues. Causal factors must always be investigated.

Examples of relevant SAEs:

- Severe OHSS requiring hospitalization
- Bleeding and/or infection in relation to the oocyte pick-up

Examples of adverse events with a possible impact on the outcome of the investigation

- embryos transferred to the wrong patient
- loss of gametes/embryos resulting in total/decreased loss of chance of pregnancy in one cycle, such as
    - technical failure of incubator or cryotank

    - accident with culture dishes,
    - embryos discarded by mistake
    - embryos lost due to microbial contamination
- labelling error of tubes/dishes containing the oocytes/sperm/embryos
- mix-up of sperm samples during preparation/treatment
- sperm sample contaminated by another sample (e.g. with a used pipette)
- oocytes fertilised with spermatozoa from the wrong person

Examples of device deficiencies
- No score due to low-quality images or air bubbles
- Cannot access the software for unknown reasons

16.6 Summary of registering and reporting events

Type of event	Registered	Reported
Adverse event	Yes, if it has been identified in the CIP as being critical to the evaluation of the results of the clinical investigation.	No
Serious adverse event	Yes	Yes, if a causal relationship with the investigational device, the comparator or the investigation procedure exists or is reasonably possible.
Device deficiency	Yes, if it might have led to a SAE if appropriate action had not been taken, intervention had not occurred, or circumstances had been less fortunate.	Yes, if it might have led to a SAE if appropriate action had not been taken, intervention had not occurred, or circumstances had been less fortunate.
New findings of any of the above	Yes	Yes, if reported, otherwise no.

16.7 Emergency contacts for reporting SAEs
SAEs are reported from within the eCRF. Any SAEs reported via the eCRF will also be handled under the post-market surveillance/vigilance system.
In case of ambiguities, the clinical investigations team can be contacted by email: clinicalinvestigations@vitrolife.com

17 PUBLICATION POLICY

The clinical investigation is registered in a publicly accessibly database (see the synopsis table). The results of the investigation will be made publicly available and offered for publication in scientific journals regardless of the outcome.

The personal data of participants will remain confidential. Study reports will not contain any information that can identify participants. However, the sponsor's monitor or representative and regulatory representatives, auditors and inspectors may need access to medical records to verify the authenticity of collected data.

17.1 Main results

- The primary results of this RCT will be announced in one of the major conferences in the field of Reproductive Medicine.
- The main publication reporting the results of the primary outcome (first phase of the trial) will be submitted for publication in a journal of high impact factor.

To evaluate the contribution that any effect makes to clinical care by:
Measuring the number of cycles where iDAScore affects the decision about which embryo is to be transferred in the first embryo transfer.

17.2 Ethical oversight

The study will be overseen by the relevant institutional ethics committee. Ethical approval for the study has been obtained for an earlier version of this study protocol (version 20) of this protocol from several Ethics committees. Further approval for this version will be sought.

18 REFERENCES

Adolfsson E, Porath S, Andershed AN. External validation of a time-lapse model; a retrospective study comparing embryo evaluation using a morphokinetic model to standard morphology with live birth as endpoint. JBRA Assist Reprod. 2018. **22** (3): 205-214

Gardner DK, Lane M, Stevens J, Schlenker T, Schoolcraft WB. Blastocyst score affects implantation and pregnancy outcome: towards a single blastocyst transfer. Fertil Steril. 2000. **73** (6):1155-8.

Goodman LR, Goldberg J, Falcone T, Austin C, Desai N. Does the addition of time-lapse morphokinetics in the selection of embryos for transfer improve pregnancy rates? A randomized controlled trial. Fertil Steril. 2016.**105** (2):275-85.

Rubio I, Galán A, Larreategui Z, Ayerdi F, Bellver J, Herrero J, Meseguer M. Clinical validation of embryo culture and selection by morphokinetic analysis: a randomized, controlled trial of the EmbryoScope. Fertil Steril. 2014.**102** (5):1287-1294.

Simon C, Sakkas D, Gardner DK, Critchley HO. Biomarkers in reproductive medicine: the quest for new answers. Hum Reprod Update. 2015. **21** (6):695-7.

Tran A, Cooke S, Illingworth PJ, Gardner DK. Deep learning as a predictive tool for fetal heart pregnancy following time-lapse incubation and blastocyst transfer. 2019. Hum Reprod. **34** (6), 1011–1018.

Mehta CR, Pocock SJ. Adaptive increase in sample size when interim results are promising: a practical guide with examples. Statistics in medicine. 2011. **30** (28): 3267-3284.https://www.ncbi.nlm.nih.gov/pubmed/22105690

Bendus. A, Mayer. J, Shipley. S.K., and Catherino, W.H. Interobserver and intraobserver variation in day 3 embryo grading. Fertil. Steril. 2006. **86** (6):

Paternot. G., Wetzels, A.M., Thonon. F., Vansteenbrugge, A., Willemen, D., Devroe, J., Debrock, S., D'Hooghe, T.M. and Spiessens, C. Intra- and interobserver analysis in the morphological assessment of early stage embryos during an IVF procedure: a multicentre study. 2011. Reprod. Biol. and Endocrinol., **9**: 127.

APPENDIX 1. SCORING OF DAY 5-6 EMBRYOS

This procedure details:

- The Blastocyst Stage Grading that is to occur in all Laboratories.
- This process cannot be duplicated/paraphrased or copied into state quality system documents.
- The state laboratory policies/work instructions must refer back to this document whenever Cleavage Stage Grading occurs.

Responsibility:

- Documents has been prepared by agreement between all Scientific Directors, and can only be changed by agreement of these Scientific Directors

Expected Development stages of Embryos-Blastocysts:

- Day 2 Embryos: 2 to 6 cells.
- Day 3 Embryos: >=6 cells. May also be compacting (early morula).
- Day 4 Embryos: early morula (compacting) or morula. It is also not unusual to see 10 and 12 cell embryos at this stage, or very early blastocysts (cavitating morula).
- Day 5 Embryos: morula, early blastocyst, blastocyst, expanded blastocyst, hatching blastocyst, or hatched blastocyst.

Blastocyst stage grading:

1. Blastocyst stage grading is based on a 3-stage system incorporating the degree of expansion, Trophectoderm status, and Inner Cell Mass status.

2. On D5 and D6, there is an increasing Pregnancy rate as the Gardner scale increases (0-to-6).

3. The recommendation is that D5 and D6 blastocysts with any combination of ICM or Trophectoderm quality grading of "C", will not be transferred or cryopreserved, except under the specific direction of the managing physician:

As defined on D5 (114-118hpi), or D6 (138-142hpi)		
Exp. Grade	Development Stage	Definition
0	Cleavage Stage	Still at Cleavage stage. Blastomeres visible.
C	Compacting	Cell membranes/borders becoming undefined.
M	Morula	Full compaction, blastomeres not clearly visible. No cavitation visible.
1	Cavitating	Blastocoel takes up less than half the space of the embryo.
2	Early Blastocyst	Blastocoel takes up greater than, or equal to half the space of the embryo.
3	Blastocyst	Blastocoel expanded into the entire volume of the embryo, pressing the Trophectoderm cells tightly against the inside of the zona pellucida.
4	Expanded blastocyst	Blastocoel volume larger than that of the early embryo, zona pellucida is thinning.
5	Hatching Blastocyst	Blastocyst is beginning to herniate out of the zona pellucida.
6	Hatched Blastocyst	Blastocyst has completely hatched out of the zona pellucida.
D	Degenerate	Majority of cells show signs of necrosis, apoptosis.
P	Pulsing / Collapsed	Blastocyst which had been expanded, but upon assessment has collapsed, or 'pulsed' so that the cells are pulled away from the zona.

Inner Cell Mass Grading (Blasts at stage 3,4,5,6)		
Gardner ICM	Quality	Definition
A	Good	Many cells, tightly packed
B	Fair	Several cells, loosely grouped
C	Poor	Very few cells

Trophectoderm Grading (Blasts at stage 3,4,5,6)		
Gardner Trophectoderm	Quality	Definition
A	Good	Many cells forming a cohesive epithelium
B	Fair	Continuous but irregular cells
C	Poor	Patchy epithelium with few cells

Ranking guideline for Group A (standard morphologic criteria) for Day 5 fresh embryo transfers:

Ranking guidelines	
Order	1. Developmental stage 6 > 5 > 4 > 3<br>2. TE grade A > B<br>3. ICM grade A > B<br>4. Developmental stage 2 > 1<br>5. Any combination of ICM or Trophectoderm quality grading of "C", or embryos with developmental stage graded as "Morula" or below, will not be transferred or cryopreserved, except under the specific direction of the managing physician.
Examples	A blastocyst graded as '5BB' will be chosen over '4AA'<br>A blastocyst graded as '4BA' will be chosen over '4AB'<br>A blastocyst graded as '2' will be chosen over '4BC'

Ranking guideline for Group A (standard morphologic criteria) for frozen embryo transfers:

Ranking guidelines	
Principles	The warming of embryos will proceed on the ranking guidelines across embryos vitrified on Day 5 or 6<br>The first embryo to be warmed will be the one with the highest morphological grading, followed by day of vitrification
Order	1. Developmental stage 6 > 5 > 4 > 3<br>2. TE grade A > B<br>3. ICM grade A > B<br>4. Day 5 > Day 6<br>5. If vitrified, any blastocyst combination of ICM or Trophectoderm quality grading of "C" will be warmed last
Examples	A Day 6 blastocyst graded as '5AA' will be chosen over a Day 5 '4AA'<br>A Day 5 blastocyst graded as '4AA' will be chosen over a Day 6 '4AA'<br>A Day 6 blastocyst graded as '4BA' will be chosen over Day 5 blastocyst '4AB'

Statistical Analysis Plan

eValuating iDA Selection Ability. The VISA study.

Will embryo selection through use of artificial intelligence (iDA) perform equally compared to blastocyst scoring?

2022-11-06

Author

Name/Title:
Nils-Gunnar Pehrsson / Senior Statistician, CEO, Statistiska Konsultgruppen

Nils-G. Pehrsson .... 8 Nov 2022

Signature Date

Approvals

Name/Title:
Peter Illingworth / Principal Investigator, Virtus Health

[illegible] ....

Signature Date 9/11/22

Name/Title:
Thorir Hardarson / Vitrolife AB

[illegible] ....

Signature Date [illegible]

Revisions

Version	Description of Changes	Date
1	A general overview and adjustments according to the latest CIP version (Sweden)	2022-08-17
2	10 clinics in 2 countries have been added	2022-08-17
3	Changes in which baseline parameters should be used	2022-08-17
4		

Table of Contents

1 Study Details ... 7
Study Objectives ... 7
1. Live birth rate ... 7
2. Positive hCG rate ... 7
3. Rate of non-viable intrauterine pregnancies ... 7
4. Ongoing pregnancy rate in patients with maternal age above 35 ... 7
Study Design ... 7
Treatment Groups ... 8
Sample Size calculation ... 9
2 Study Populations ... 10
Definition of Study Populations ... 10
2.1.1 Intent-to-Treat Population ... 10
2.1.2 Full Analysis Set ... 10
2.1.3 Per-Protocol Population ... 10
2.1.4 Safety Population ... 10
3 Poolability of Investigative sites ... 11
4 Study Variables ... 12
Baseline Variables ... 12
4.1.1 Demographics and Baseline Characteristics ... 12
Treatment variables ... 12
Efficacy Variables ... 13
4.1.2 Primary Efficacy Variable ... 13
4.1.3 Secondary efficacy Variables ... 13
Safety Variables ... 14
4.1.4 Adverse Events (AE) ... 14
5 Statistical Methodology ... 14
General Statistical Methodology ... 14
Patient Disposition and Data Sets Analysed ... 16
Protocol Violations/Deviations ... 16
Demographics and Baseline Characteristics ... 16
Main Treatment variables ... 17
Efficacy Analyses ... 17

5.1.1 Secondary Efficacy Analyses 19
5.1.2 Exploratory Efficacy analyses 19
5.1.3 Pre-specified subgroup analyses 19
5.1.4 Exploratory interaction analyses 19
Safety Analyses 21
5.1.5 Adverse Events 21
6 DSMB analyses and Interim Analyses 21
Data Safety Monitoring Board analyses. 21
Interim analysis 22
7 Changes of Analysis from Protocol 22
8 Listing of Tables and Listings 22
Listing of Tables 22
Listing of graphs 23
Listing of Listings 24
9 References: 25

LIST OF ABBREVIATIONS

Abbreviation	Definition
FHB	Fetal heartbeat
iDA	iDAScore® which is the commercial name for the deep learning tool investigated
hCG	Human chorionic gonadotropin
IU	International units
Hpi	Hours post insemination
CIP	Clinical investigation protocol
RCT	Randomised controlled trial
CI	Confidence interval
ITT	Intention-to-treat
FAS	Full analysis dataset
PP	Per-protocol
BMI	Body mass index
FSH	Follicle stimulation hormone
LH	Luteinizing hormone
PN	Pronuclei
GnRH	Gonadotropin-releasing hormone
ICSI	Intracytoplasmic sperm injection
IVF	In-vitro fertilisation
AI	Artificial intelligence
AE	Adverse event
OR	Odds ratio
RR	Relative risk

SAE	Serious adverse event
DSMB	Data safety and monitoring board

1 STUDY DETAILS

Study Objectives

The primary objective for this study is to investigate whether selection of a single blastocyst for transfer using the deep learning-based support tool called iDAScore results in a non-inferior clinical pregnancy rate compared to when the selection is performed by trained embryologists using conventional morphology only. Clinical pregnancy is defined in this study as the detection of a fetal heartbeat (FHB) by ultrasound after 42 days of gestation (week 6) (including ectopic pregnancies, as per the ICMART definition).

The secondary objectives for this study are:

To investigate whether blastocyst selection supported by iDAScore results in non-inferior rates of the below parameters compared to when embryo selection performed by trained embryologists using conventional morphology only:

1. Live birth rate* (defined as the number of patients with at least one live birth after 22 completed weeks of gestation. If the exact gestational age is not known then a birth weight of ≥500gr can be used as a cut-off.

2. Positive β-hCG rate (defined as the number of patients with a positive β-hCG, determined by a hCG measurement from a blood sample or using urinary sticks).

3. Rate of non-viable pregnancies (defined as the difference between number of clinical pregnancies (excluding ectopic pregnancies) and number of positive β-hCG pregnancies)

4. Ongoing pregnancy rate* (defined as the number of patients with a viable pregnancy at ≥12 weeks of gestation)

*These secondary parameters will not be included in the first statistical analysis and report as these will not be available until approximately 12 months after the initial data collection.

Study Design

This is a non-inferiority, prospective parallel group, double blind, randomized controlled trial in 15 IVF centres in 4 countries (Australia, Denmark, Sweden and United Kingdom).

Treatment Groups

Patients will be randomly allocated (1:1) to two different study groups if at least two embryos have developed to the blastocysts stage (Gardner score 2 or higher):

A. **Control group: Embryo selection by standard morphologic criteria.**

- The embryo for transfer will be selected by the embryologist based on the morphologic appearances on day 5, according to the Gardner criteria (Gardner et al., 2000) using the ranking guideline (Appendix 1, in CIP).
- Regardless of whether a transfer takes place any embryos that fulfil the criteria applied in the laboratory for cryopreservation will be cryopreserved (Appendix 1 in the clinical investigation protocol (CIP)). If there is doubt about whether an embryo is suitable for cryopreservation, embryos may be held over to day 6 and the decision will be made then.
- The order of rewarming and transfer of cryopreserved embryos for later transfer will be made according to Appendix 1 in the CIP based on the all the information that is available by day 6.

B. **Treatment group: Embryo selection supported by iDAScore**.

- The time-lapse videos will be analysed by iDA at 114-118 hours post insemination (hpi) and the embryo with the highest iDA score will be selected for fresh transfer on day 5.
- Any remaining viable embryos in this group will be cryopreserved if they have either:

  - reached Gardner Grade 3 or beyond AND would normally be cryopreserved or;
  - have reached Gardner Grade 3 AND achieved a score on iDA of 5 or more.
- All other embryos will be cultured until day 6, re-scored at 138-142 hpi and according to the above criteria.
- The warming and transfer of embryos will be performed based on the ranking of the iDA score across the two days. The first embryo to be warmed will be the one with the highest iDAScore. If this embryo does not survive warming and is not suitable for transfer, the next embryo to be warmed will be selected based on the iDAScore, until an embryo is warmed and is suitable for transfer.

Embryo transfer will be performed using the clinic's routine methods. Luteal support will be administered using the standard protocol of each clinic. Both the treating clinician and the patient will remain blinded to the randomization outcome until after the first embryo transfer has been completed.

Sample Size calculation

It is estimated from the results in clinics that clinical pregnancy is estimated to be 35.4% for trained embryologists. If non-inferiority margin is defined as - 5%, the lower limit of the two-sided 95% confidence interval (CI) for the difference between iDAScore group and Trained embryologist group shall not be less than -5% with a probability of 90% (β=10%), with an estimation of 5% or more clinical pregnancies in iDAScore group, 494 women per randomization group is needed to show non- inferiority with two-sided Farrington-Manning test. For protection against a 5% loss to follow-up, 1040 patients in total, 520 per group, are needed for recruitment.

2 STUDY POPULATIONS

Definition of Study Populations

2.1.1 Intent-to-Treat Population

All randomized subjects will be included in the Intent-to-Treat (ITT) population. Subjects will be analysed according to randomized group.

2.1.2 Full Analysis Set

All randomized subjects with measurement of primary efficacy variable will be included in the Full Analysis Set (FAS). Subjects will be analysed according to randomized group.

2.1.3 Per-Protocol Population

All randomized subjects with no significant protocol violations will be included in the Per Protocol (PP) population. Subjects will be analysed according to actual embryo selection method. The Per Protocol population will be defined during the clean file meeting before the database lock without knowledge to which randomised group the patient belongs to.

2.1.4 Safety Population

All enrolled subjects who started one of the study embryo selections methods (i.e., in patients with a minimum of two early blastocysts) will be included in the safety population.

The final decisions regarding all the above study population will be taken at the Clean File meeting before the database lock.

3 POOLABILITY OF INVESTIGATIVE SITES

The data from all investigative sites will be pooled based on the assumption of clinical comparability: the sites used a common protocol; the sponsor adequately monitored the study to assure protocol compliance; and the data gathering and validation mechanisms were the same across all study sites.

Analyses to justify pooling will include the following:

- The primary endpoint will be presented by site: Mean difference in percentages of clinical pregnancy with 95% CI.

- The justification for pooling all the data to estimate a common effect across study sites requires the homogeneity of response across study sites. An exact Pearson Chi-square test of the proportions of clinical pregnancies over sites will be generated to test whether the investigational sites differ with respect to primary study on the ITT population (only the site will be included in this analysis model). The test of homogeneity of response will be based on a two-sided significance test at the 0.10 level of significance.

- If the sites differ by this test then a second analysis will be done including study site and all baseline characteristics that have a $p<0.10$ in the analysis of baseline variables by site to understand if the imbalance in the primary endpoint between sites is related to an imbalance in baseline characteristic.

The analysis of study sites may require the formation of pseudo-sites because the small study sites will not provide appropriate information to allow the analysis above. Study sites with fewer the 16 subjects will be

combined into pseudo-sites for the poolability analysis using the following method. The smallest study site with less than 16 subjects will be combined with the next smallest study site. These two sites may be combined with a third site if the combined number of patients remain less than 16.

4 STUDY VARIABLES

Baseline Variables

4.1.1 Demographics and Baseline Characteristics

1. Age (maternal*/paternal), continuous variables.
2. Reason for infertility (couple), categorical variable.
3. Height and weight (mat.), continuous variables
4. BMI (mat.), continuous variable
5. Type of menstruation (mat.), dichotomous variable
6. Number of previous stimulated IVF cycles leading to oocyte pick-up (couple)*, ordered categorical variable (7 categories)
7. Previous pregnancies in current relationship, ordered categorical variable (5 categories)

Treatment variables

1. FSH starting dosage, continuous variable
2. FSH total dosage, continuous variable
3. GnRH downregulation (agonist/ antagonist)
4. Source of sperm, categorical variable (6 categories)
5. Duration of ovarian stimulation, continuous variable.
6. Number of oocytes*, both as continuous variable and ordered categorical variable (7 categories)

7. Method of fertilization (ICSI/Standard IVF/Combined)* Categorical variable
8. Number of normally fertilized oocytes (2PN)*, continuous variable
9. Number of blastocysts at Gardner Scale 2 or beyond by day 5 (Early blastocysts), continuous variable
10. Proportion of cycles where the embryo selected by the embryologist had the highest iDAScore (Only in the iDAScore group).
11. Number of cryopreserved embryos on day 5 and 6, continuous variable
12. Morphological score of the transferred embryo, continuous variable
13. iDAScore® (treatment group) of the transferred embryo, continuous variable
14. Type (categorical variable) and duration (continuous variable) of luteal phase support

*) These variables are used in a deterministic minimization procedure together with center during randomization. All variables are entered as Categorical variables: Age (5 cat.), number of earlier IVF cycles (7 cat.), number of oocytes (7 categories), fertilization method (3 cat.), number of 2PN oocytes (7 categories) and center (14-15 centers).

Efficacy Variables

4.1.2 Primary Efficacy Variable

Primary efficacy variable will be Clinical pregnancy with fetal heartbeat after the first embryo transfer per randomised patient. If no fresh nor frozen embryo can be transferred, then for the ITT population a negative clinical pregnancy is given for these patients.

4.1.3 Secondary efficacy Variables

Secondary efficacy variables will be the following outcome variables:

- Live birth (Y/N). Will be analysed in a second database lock.
  With pass criteria non-inferiority analysis, non-inferiority margin -5%.

- Positive hCG rate per randomized patient
  With pass criteria non-inferiority analysis, non-inferiority margin -5%.
- Non-viable intrauterine pregnancies

Safety Variables

4.1.4 Adverse Events (AE)

The occurrence of adverse events is documented in the eCRF through a separate AE module.

5 STATISTICAL METHODOLOGY

General Statistical Methodology

Primary and all secondary analyses will be performed on ITT population. Complementary analyses will be performed on full analysis set (FAS) and on the per-protocol (PP) population. The primary statistical analyses will be calculation of the mean percentage difference with two-sided 95% confidence interval (CI) regarding the primary efficacy variable Clinical Pregnancy with fetal heartbeat after the first embryo transfer between the iDA group and the trained embryologist (standard) group unadjusted with Farrington-Manning 95% CI. If the lower limit of this 95% CI is larger than -5%, the non-inferiority margin, then non-inferiority is achieved. If non-inferiority is achieved, then we will test primary efficacy variable for superiority with two-sided Fisher's exact test.

Unadjusted and adjusted relative risk (RR) between the two groups with 95% CI will be calculated with Poisson regression model with a robust error variance, (see reference 2) for primary and secondary efficacy variables.

Regarding multiplicity for the primary analysis (clinical pregnancy) and the first two secondary analyses (live birth) and Positive hCG a fixed – sequence test will be applied. (see reference 3)

If non-inferiority is achieved in primary analysis then the probability mass from the primary analysis will be transferred to the first secondary analysis, live birth.

If non-inferiority is achieved also for live birth then the non-inferiority result for live birth will also be confirmative, and the probability mass will be transferred to the second secondary analysis of positive hCG rate. If non-inferiority is achieved also for positive hCG rate birth, then the non-inferiority results for positive hCG rate will also be confirmative

For the other secondary variables, the p-values will be given for descriptive purpose and no multiplicity adjustment will be performed. These analyses will be considered exploratory.

For unadjusted comparison between the two randomized groups the following tests will be performed: Fisher's exact test for dichotomous variables,

Fisher's non-parametric permutation test for the mean difference between two independent samples for continuous variables,

Mantel-Haenszel chi-square test for ordered categorical variables and

Pearson chi-square test for non-ordered categorical variables.

Dichotomous data will be expressed as numbers and percentages. Continuous variables will be described with mean, standard deviation, median, quartile 25%, quartile 75%, minimum and maximum.

If baseline confounders, variables that differ statistically and clinically between the randomized groups and known to predict primary outcome variable, are found then complementary analyses will be performed adjusted for these baseline variables.

All tests will be two-tailed and conducted at 0.05 significance level.

All safety analyses will be descriptive by performed embryo selection method.

All analyses will be performed by using SAS® v9.4 (Cary, NC).

Patient Disposition and Data Sets Analysed

The number of subjects included in each of the ITT, FAS, PP and safety populations will be summarized for each treatment group and overall. The number and percentage of subjects randomized and treated will be presented. Subjects who completed the study and subjects who withdrew from study prematurely will also be presented with a breakdown of the reasons for withdrawal by treatment group for the ITT, FAS, PP and safety populations.

Protocol Violations/Deviations

Major protocol deviations are those that are considered to influence the analysis. A list of protocol deviations that were anticipated before the start of the trial was generated. Once the data collection has been finalized and before database lock the steering committee will review all the possible deviations and if needed create additional categories.

The number of patients with major protocol deviations will be summarized per treatment group.

Demographics and Baseline Characteristics

Demographics and baseline characteristics will be summarized by treatment group for the ITT, FAS and PP populations and analysed according to the methods described in section "General Statistical Methodology" above.

Main Treatment variables

Main treatment variables will be summarized by treatment group for the FAS and PP populations and analysed according to the methods described in section "General Statistical Methodology" above.

Efficacy Analyses

Primary Efficacy Analysis

The primary statistical analyses will be calculation of the mean percentage difference with two-sided 95% confidence interval (CI) regarding the primary efficacy variable Clinical Pregnancy with fetal heartbeat after the first embryo transfer between the iDA group and the trained embryologist (standard) group unadjusted with Farrington-Manning 95% CI. If the lower limit of this 95% CI is larger than -5%, the non-inferiority margin, then non-inferiority is achieved. This analysis will be applied on the ITT population (primary) and PP population (sensitivity analysis). Any inconsistencies in these analyses will be discussed in the study report. Unadjusted relative risk (RR) with 95% CI will also be calculated. The Clinical pregnancy rate with exact 95% CI will be calculated for each of the randomised groups.

The following sensitivity analysis will also be performed adjusted for centre and stratification (minimization) variables:

To account for the lack of independence introduced by both the potential clustering within centers and the optimal randomization process (minimization), we will also estimate the difference in the clinical pregnancy probability between the two randomized groups with a 95% CI using a linear mixed effects regression model (with identity link function) with center as a random effect and fixed effects for the following variables used in the randomization:

- <u>Woman's age:</u>

< 25; 25 <= Age < 30; 30 <= Age < 35; 35 <= Age < 40; 5. 40 <= Age

- Number of previous stimulated IVF cycles leading to oocyte pick-up

1-5; 6 – 10; 11 – 15; 16 – 20; 21 – 25; 26 -> ....

- Number of oocytes:
- *2 – 5; 6 – 10; 11 – 15; 16 – 20; 21 - 25; 26 -> ....*
- Fertilization method:

IVF for all; ICSI for all; ICSI for some

- Number of 2PN oocytes
- *2 – 5; 6 – 10; 11 – 15; 16 – 20; 21 - 25; 26 -> ....*
- Center.
  Adjustment with centre as random effect, fertilization method as class variable and the other four as continuous variables.

To account for potential non-constant variance of errors in this model, we will estimate the empirical covariance matrix using the heteroskedasticity consistent method (HC3) as proposed by MacKinnon and White (see reference 1).

Adjusted relative risk (RR) with 95% CI will also be calculated adjusted for the minimization variables using Poisson regression model with a robust error variance. .

If non-inferiority is achieved, then we will test the primary efficacy variable for superiority with a two-sided Fisher's exact test. The primary analysis will also be performed per center. If baseline confounders are found the adjusted analyses will also be performed adjusted for these variables.

5.1.1 Secondary Efficacy Analyses

The secondary efficacy analyses will be the analysis of the secondary efficacy variables defined in section 4.1.3 "Secondary efficacy variables" according to the methods given in section 5, under "General Statistical Methodology".

5.1.2 Exploratory Efficacy analyses

- Compare the clinical pregnancy in the iDAScore groups that doesn't agree with the embryologist with clinical pregnancy in the agreement group in the iDAScore arm, with the same analysis as the primary analysis.
- Calculate the percent agreement in the iDAScore arm between the iDAScore and the embryologist regarding clinical pregnancy.

5.1.3 *Pre-specified subgroup analyses*

Pre-specified subgroup analysis will be performed on the primary and important secondary variables between the two randomized groups for the following baseline subgroup:

- Women older than 35 years.

5.1.4 Exploratory interaction analyses

Exploratory interaction analyses between the two randomized group and the following baseline variables regarding analyses of primary and selected secondary variables:

- Maternal age
- Number of blastocysts at Gardner Scale 2 or beyond by day 5
- Freeze all

Model: Outcome = Randomized_Group Baseline_var Randomized_Group *Baseline_var

These analyses should be performed with no other adjustments with Poisson regression model with a robust error variance.

If interaction p-value <0.10 then subgroups analysis will follow regarding primary and selected secondary analyses in subgroups of the baseline variable.

Statistical Analysis Plan (SAP) for the sub study:

Comparing time used for embryo evaluation between the conventional morphology group (control) and iDAScore (treatment) group.

Fisher's non-parametric permutation test for the mean difference between two paired samples will be used to analyze time used for embryo evaluation between the two methods used on the same individuals. For each method mean, SD, Median, minimum and maximum will be given. For the difference between the two methods mean with 95% CI, SD, median, minimum and maximum will be given. Six subjects are the minimum number to be included from each centre.

Separate analyses will also be performed for each site and divided in three categories of number of embryos.

Follow-up for primary efficacy analysis

All patients undergoing a freeze-all cycle for any clinical indication, are expected to return for an embryo transfer within 3 months after their oocyte collection. All freeze-all patients that have not returned for their first rewarmed blastocyst 3 months after the last patient was randomized will be treated as a protocol violations.

Safety Analyses

5.1.5 Adverse Events

Only treatment-emergent AEs will be included in the summaries for safety population.

A summary of subjects reporting at least one of the following AEs will be presented in an overview table:

- Any AE
- Any SAE
- Any treatment-related AE
- Any treatment-related SAE
- Any AE leading to discontinuation
- *Any device related events*
- Death

Summaries per SOC and PT presenting n (%) of AEs and n (%) of subjects with at least one AE will be provided for:

- All AEs (includes all serious and non-serious AEs)
- All AEs by maximum reported intensity
- All AEs by causality
- All SAEs
- All AEs leading to discontinuation

6 DSMB ANALYSES AND INTERIM ANALYSES

Data Safety Monitoring Board analyses.

An independent DSMB has been appointed to follow the safety and efficacy monitoring as well as the overall conduct of the study. The board consists of a statistician and a medically knowledgeable person

unrelated to the study. The role of the DSMB has been set out in a separate charter and the members will hold regular meetings where the study efficacy and safety will be assessed and if necessary, suggestions for changes in study protocol. For early termination of efficacy for benefit (clinical pregnancy substantially higher in iDA group than in the trained embryologist group) the DSMB should use O'Brian-Fleming's sequential boundaries on the positive side. The DSMB should start to look at efficacy data for benefit after 50% of the subjects have completed evaluation of the primary outcome.

For early termination for harm (clinical pregnancy substantially lower in iDA group than in the trained embryologist group) the DSMB should use a Z value of -2.4 and perform first analysis when 20% of subjects have completed evaluation of the primary outcome. All interim analyses will be performed by the DSMB and will be strictly blinded for everybody outside DSMB.

Interim analysis

No other interim analysis will be performed.

7 CHANGES OF ANALYSIS FROM PROTOCOL

No major changes, only more details of the analyses have been added.

8 LISTING OF TABLES AND LISTINGS

Listing of Tables

Table Number	Table Title
14.1.1	Patient Disposition and Data Sets Analysed (ITT Population)
14.1.2	Protocol Deviations Leading to Exclusion from PP Population (ITT Population)
14.1.3.1	Demographics and Baseline Characteristics (ITT Population)
14.1.3.2	Demographics and Baseline Characteristics (FAS Population)

14.1.3.3	Demographics and Baseline Characteristics (PP Population)
14.1.3.4	Main Treatment Variables (ITT Population)
14.1.3.5	Main Treatment Variables (FAS Population)
14.1.3.6	Main Treatment Variables (PP Population)
14.1.4.1	Concomitant Medications (FAS population)
14.2.1.1	Primary Efficacy Analysis (ITT Population)
14.2.1.2	Primary Efficacy Analysis (PP Population)
14.2.1.3	Primary Efficacy Analysis Sensitivity Analyses, adjusted for centre and selected allocation variables (ITT Population)
14.2.1.5	Primary Efficacy Analysis for each centre (ITT Population)
14.2.1.6	Poolability of investigative sites
14.2.2.1	Secondary Efficacy Analysis (FAS Population)
14.2.2.2	Secondary Efficacy Analysis (PP Population)
14.2.3.1	Subgroup Analyses
14.2.3.2	Exploratory Interaction analyses (FAS Population)
14.2.3.3	Results from study: Time evaluation
14.3.2.1	Summary of Adverse Events (Safety Population)
14.3.2.2	All Adverse Events
14.3.2.3	All Adverse Events by maximum reported intensity
14.3.2.4	All Adverse Events by causality
14.3.2.5	All Serious Adverse Events
14.3.2.6	All Adverse Events leading to discontinuation

Listing of graphs

15.1	Figure 1 should be the flow chart of Screening, Eligibility Assessment, randomisation, ITT, PP analysis as well (i.e. CONSORT Flow Chart)
15.2	Percent of Clinical Pregnancy, Biochemical Pregnancy, and Number of sacs with 95% CI for iDAscore and control group. (ITT population). Vertical bar charts with 95% CI
15.3	Unadjusted mean difference with 95% CI between iDAscore and control group regarding Clinical Pregnancy, Biochemical Pregnancy and Number of sacs. (ITT population)<br>Vertical bar charts with 95% CI
15.4	Adjusted mean difference with 95% CI between iDAscore and control group regarding Clinical Pregnancy, Biochemical Pregnancy and Number of sacs. (ITT population)<br>Vertical bar charts with 95% CI
15.5-15.7	The same figures as in 15.2 to 15.4 for Live birth
15.5	Figures regarding distribution of time to evaluation between the two groups and within each group .

	Results from the substudy. (ITT population)<br>Box plots with means.

Listing of Listings

Listing number	Listing Title
16.2.1	Discontinued Patients
16.2.2	Patients with Important Protocol Deviations
16.2.3	Patients Excluded from the Efficacy Analysis
16.2.4.1	Demographics and Baseline Characteristics
16.2.4.2	Main Treatment variables
16.2.4.3	Medical History
16.2.4.3	Prior and Concomitant Medications
16.2.5	Efficacy Variables
16.2.6	Adverse Events

REFERENCES:

1. MacKinnon, J. G., and White, H. (1985). "Some Heteroskedasticity-Consistent Covariance Matrix Estimators with Improved Finite Sample Properties." *Journal of Econometrics* 29:305–325.

2. Guangyong Zou **(**2004**).**" A Modified Poisson Regression Approach to Prospective Studies with Binary Data". *American Journal of Epidemiology, Volume 159, Issue 7, Pages 702–706,*

3. A. Dmitrienko, R. B. D'Agostino, (2018). "Multiplicity Considerations in Clinical Trials". New England Journal of Medicine 378;22
