# Supplementary material for: Deep learning versus manual morphology-based embryo selection in IVF: a randomized, double-blind noninferiority trial
Source: Nat Med. 2024 Aug 9;30(11):3114–20. doi: 10.1038/s41591-024-03166-5 (PMC11564097; doi:10.1038/s41591-024-03166-5)
Supplement: Supplementary file 2 — Reporting Summary [file 41591_2024_3166_MOESM2_ESM.pdf]

nature portfolio

Corresponding author(s): Peter Illingworth

Last updated by author(s): Feb 12, 2024

Reporting Summary

Nature Portfolio wishes to improve the reproducibility of the work that we publish. This form provides structure for consistency and transparency in reporting. For further information on Nature Portfolio policies, see our Editorial Policies and the Editorial Policy Checklist.

Statistics

For all statistical analyses, confirm that the following items are present in the figure legend, table legend, main text, or Methods section.

n/a	Confirmed	
☐	☒	The exact sample size (*n*) for each experimental group/condition, given as a discrete number and unit of measurement
☐	☒	A statement on whether measurements were taken from distinct samples or whether the same sample was measured repeatedly
☐	☒	The statistical test(s) used AND whether they are one- or two-sided<br>*Only common tests should be described solely by name; describe more complex techniques in the Methods section.*
☐	☒	A description of all covariates tested
☐	☒	A description of any assumptions or corrections, such as tests of normality and adjustment for multiple comparisons
☐	☒	A full description of the statistical parameters including central tendency (e.g. means) or other basic estimates (e.g. regression coefficient) AND variation (e.g. standard deviation) or associated estimates of uncertainty (e.g. confidence intervals)
☐	☒	For null hypothesis testing, the test statistic (e.g. *F*, *t*, *r*) with confidence intervals, effect sizes, degrees of freedom and *P* value noted<br>*Give P values as exact values whenever suitable.*
☐	☒	For Bayesian analysis, information on the choice of priors and Markov chain Monte Carlo settings
☐	☒	For hierarchical and complex designs, identification of the appropriate level for tests and full reporting of outcomes
☒	☐	Estimates of effect sizes (e.g. Cohen's *d*, Pearson's *r*), indicating how they were calculated

Our web collection on statistics for biologists contains articles on many of the points above.

Software and code

Policy information about availability of computer code

Data collection	The image analysis was performed by iDAScore Version 1.2 (www.vitrolife.com)The iDAScore® algorithm is commercially available. It was developed by Vitrolife to be used within the EmbryoViewer workstation with EmbryoScope time-lapse incubators. The code is proprietary, but iDAScore and the necessary hardware can be obtained from Vitrolife (www.vitrolife.com).<br>All data in the study was collected using a commercial eCRF provider MariaDB v10.6 and Database Platform VI v7.1.6 by Stockholm Data Design, Sweden. The randomisation was performed within the eCRF using Web-based interactive response technology.
Data analysis	Raw analysis data was stored as csv files and analyzed using SAS v9.4 as described in detail in the Statistical Analysis Plan

For manuscripts utilizing custom algorithms or software that are central to the research but not yet described in published literature, software must be made available to editors and reviewers. We strongly encourage code deposition in a community repository (e.g. GitHub). See the Nature Portfolio guidelines for submitting code & software for further information.

Data

Policy information about availability of data

All manuscripts must include a data availability statement. This statement should provide the following information, where applicable:

- Accession codes, unique identifiers, or web links for publicly available datasets
- A description of any restrictions on data availability
- For clinical datasets or third party data, please ensure that the statement adheres to our policy

Data collected for the study, including deidentified participant data, and the data dictionary, will be made available to others. Related documents, including the study protocol, the statistical analysis plan and the informed consent forms will be available following publication. The data and documents will be made available on request to academic researchers following review by the Study steering committee (PI, CV, DG, SN, JB, ML, TH) and completion of a data sharing agreement. Requests for data sharing should be addressed to the corresponding author. Approval of requests for academic purposes will be provided within three weeks and the data supplied following approval. Approval will not be provided for commercial use of the data.

Human research participants

Policy information about studies involving human research participants and Sex and Gender in Research.

Reporting on sex and gender	This is a study of embryos. All the participants giving consent are women from whose eggs, the resulting embryos are being studied in the project and their partners (whether male or female).
Population characteristics	The study was conducted between January 2020 and September 2022. We included women undergoing IVF or ICSI after ovarian stimulation with gonadotrophins and gonadotrophin releasing hormone analogues and with the intention to treat by either transfer of a single fresh embryo on day 5 or, in a freeze-all cycle, the first rewarmed embryo. Inclusion criteria required women to be before their 42nd completed birthday with at least two early blastocysts on day 5 of embryo culture, the day of randomisation. We excluded participants if they were involved in treatment using donated eggs, intended to perform preimplantation genetic testing, used additional laboratory interventions such as intracytoplasmic morphologically selected sperm injection (IMSI), polarised light microscopy, assisted hatching, or had previously participated in the trial.
Recruitment	The study was conducted between January 2020 and September 2022. Potential participants were identified by staff members and their eligibility reviewed by one of investigator. If eligible, informed consent was sought. The resulting population, was self-selected for willingness to participate in the trial. No self-selection biases in the overall population were apparent.
Ethics oversight	IVFAustralia HREC, an HREC that is accredited with the Australian NHMRC regulating Ethics Committee.

Note that full information on the approval of the study protocol must also be provided in the manuscript.

Field-specific reporting

Please select the one below that is the best fit for your research. If you are not sure, read the appropriate sections before making your selection.

☒ Life sciences ☐ Behavioural & social sciences ☐ Ecological, evolutionary & environmental sciences

For a reference copy of the document with all sections, see nature.com/documents/nr-reporting-summary-flat.pdf

Life sciences study design

All studies must disclose on these points even when the disclosure is negative.

Sample size	The non-inferiority margin for this study was established at -5% for clinical pregnancy rate. On the basis of existing evidence from participating clinics, we expected the clinical pregnancy rate following the first embryo transfer to be 35.4%. To demonstrate with 90% power (α=0.05 & β=0.10) that the lower limit of the two-sided 95% confidence interval (CI) for the difference between the iDAScore and the standard morphology criteria group would not be less than -5%, with an expected increase in clinical pregnancy of 5% or more in the iDAScore group, we required 494 women per group. This figure was increased to 520 women per group to account for a potential 5% of loss to follow-up.
Data exclusions	No data were excluded
Replication	Not applicable to a randomised controlled trial
Randomization	The participant was allocated to treatment by randomisation through a Web-based interactive response technology (MariaDB v10.6 and Database Platform VI v7.1.6 by Stockholm Data Design, Sweden) to ensure allocation concealment.
Blinding	The embryologist was blinded to the randomisation at the time of morphology selection, by ensuring that the highest-grade morphology blastocyst was selected, using the prioritisation strategy detailed in the CIP Appendix, prior to randomisation. Both the treating clinician and

the patient remained blinded to the randomisation outcome until after the first embryo transfer.

Reporting for specific materials, systems and methods

We require information from authors about some types of materials, experimental systems and methods used in many studies. Here, indicate whether each material, system or method listed is relevant to your study. If you are not sure if a list item applies to your research, read the appropriate section before selecting a response.

Materials & experimental systems

n/a	Involved in the study
☒	☐ Antibodies
☒	☐ Eukaryotic cell lines
☒	☐ Palaeontology and archaeology
☒	☐ Animals and other organisms
☐	☒ Clinical data
☒	☐ Dual use research of concern

Methods

n/a	Involved in the study
☒	☐ ChIP-seq
☒	☐ Flow cytometry
☒	☐ MRI-based neuroimaging

Clinical data

Policy information about clinical studies
All manuscripts should comply with the ICMJE guidelines for publication of clinical research and a completed CONSORT checklist must be included with all submissions.

Clinical trial registration	Australian New Zealand Clinical Trial Registry (ANZCTR #ID 379161)
Study protocol	Attached
Data collection	The study was conducted between January 2020 and September 2022 at 14 IVF clinics in Australia and Europe
Outcomes	The primary outcome for this study was the achievement of clinical pregnancy after the first embryo transfer, whether fresh or frozen-thawed in freeze-all cycles. We defined clinical pregnancy as an intrauterine gestation with a fetal heartbeat observed after 7-9 weeks gestation. Secondary outcomes included positive hCG rate, non-viable intrauterine pregnancies, ongoing pregnancy rate (at 12 weeks gestation) and live birth rate. Non-viable intrauterine pregnancy was defined as a pregnancy where an intrauterine gestation was seen with no fetal heart found. Live birth was defined as delivery of at least one liveborn child.<br>We compared the time taken to evaluate the same embryos across three laboratories using the two assessment techniques
